# Care for capabilities: Implementing the capability approach in rehabilitation of patients with neuromuscular diseases. Study protocol of the controlled before-after ReCap-NMD study

**DOI:** 10.1371/journal.pone.0261475

**Published:** 2021-12-21

**Authors:** Bart Bloemen, Eirlys Pijpers, Edith Cup, Jan Groothuis, Baziel van Engelen, Gert Jan van der Wilt

**Affiliations:** 1 Department for Health Evidence, Donders Institute for Brain, Cognition and Behaviour, Radboud University Medical Center, Nijmegen, Netherlands; 2 Department of Rehabilitation, Donders Institute for Brain, Cognition and Behaviour, Radboud University Medical Center, Nijmegen, Netherlands; 3 Department of Neurology, Donders Institute for Brain, Cognition and Behaviour, Radboud University Medical Center, Nijmegen, Netherlands; Public Library of Science, UNITED KINGDOM

## Abstract

**Background:**

High quality care of patients with neuromuscular diseases requires a personalised approach that focuses on achieving and maintaining a level of functioning that enables them to be in a state of well-being. The capability approach states that well-being should be understood in terms of capabilities, the substantial opportunities that people have to be and do things they have reasons to value. In this Rehabilitation and Capability care for patients with Neuromuscular diseases (ReCap-NMD) study, we want to investigate whether providing care based on the capability approach (*capability care*) has an added value in the rehabilitation of patients with neuromuscular diseases (NMD).

**Methods:**

Two groups of 30 adult patients with facioscapulohumeral muscular dystrophy or myotonic dystrophy type 1 will be included. The first group will receive rehabilitation care as usual with a follow-up period of 6 months. Then, based on theory, and experiences of patients and healthcare professionals, capability care will be developed. During the following 3 months, the multidisciplinary outpatient rehabilitation care team will be trained in providing this newly developed capability care. Subsequently, the second group will receive capability care, with a follow-up period of 6 months. A mixed methods approach is used with both qualitative and quantitative outcome measures to evaluate the effect of capability care and to perform a process evaluation. The primary outcome measure will be the Canadian Occupational Performance Measure.

**Discussion:**

The ReCap-NMD study is the first study to design and implement a healthcare intervention based on the capability approach. The results of this study will expand our knowledge on how the capability approach can be applied in delivering and evaluating healthcare, and will show whether implementing such an intervention leads to a higher well-being for patients with NMD.

**Trial registration:**

Registered at Trialregister.nl (Trial NL8946) on 12^th^ of October, 2020.

## Introduction

Facioscapulohumeral muscular dystrophy (FSHD) type 1 and type 2, and myotonic dystrophy type 1 (DM1) both are slowly progressive and among the most prevalent inherited neuromuscular diseases (NMD) [1, 2]. Disease course and severity are highly variable, even within families. There is no curative treatment available for FSHD and DM1. Therefore, treatment of these patients, each with their individual phenotype, requires a personalised rehabilitation approach that focuses on achieving and maintaining a level of functioning that enables them to lead a satisfying life.

**Fig 1:**
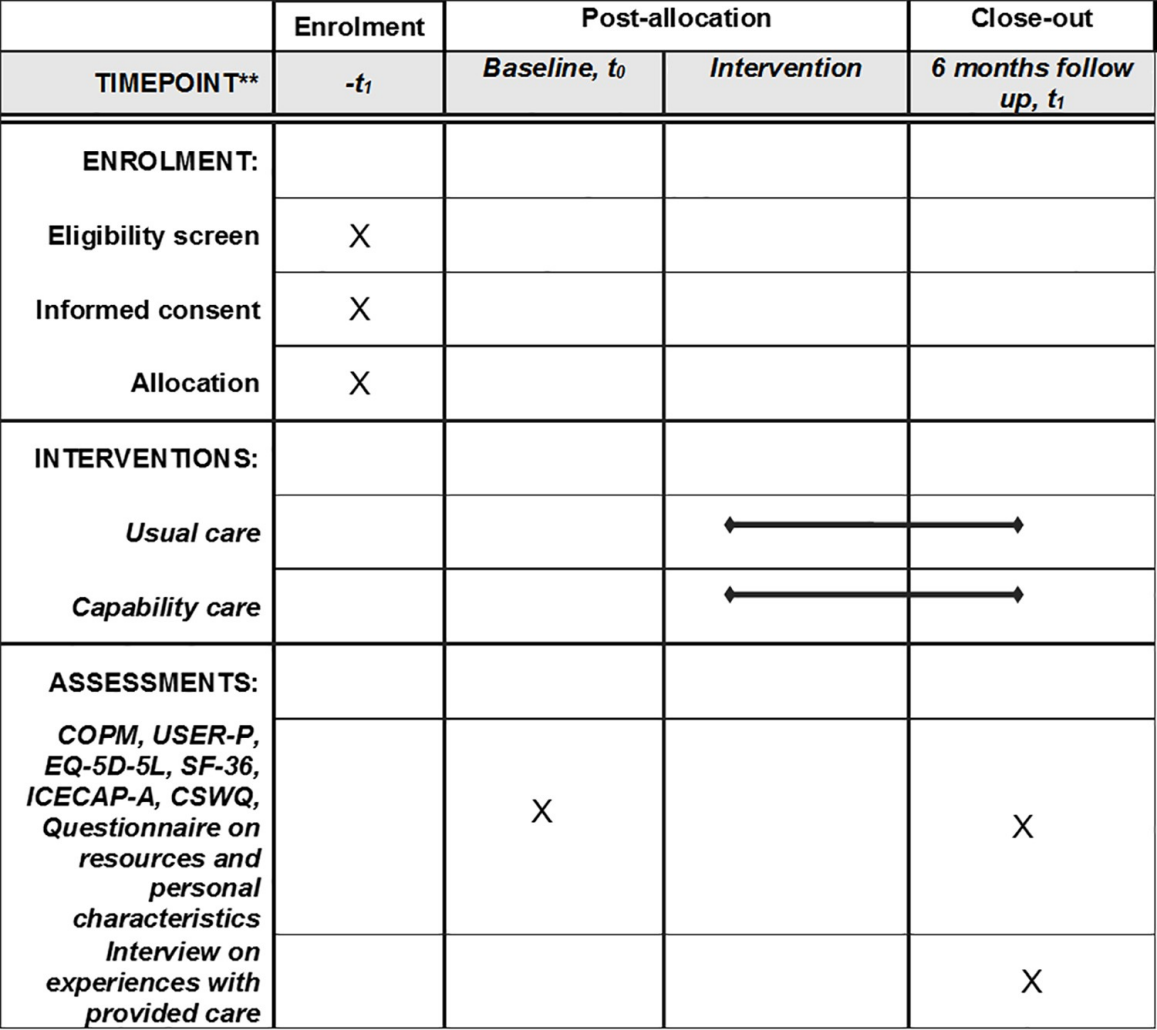
SPIRIT schedule of enrolment, interventions, and assessments.

Rehabilitation, by definition, is a process of enabling someone to live well with an impairment in the context of his or her environment and, as such, requires a complex, individually tailored approach [3]. It is aimed at optimizing body functions, activities and participation to support patients in finding a way to maintain or improve their level of functioning, leading to a higher well-being. To describe and evaluate the impact of disease on the well-being of patients, rehabilitation professionals have adopted the World Health Organisation’s (WHO) International Classification of Disability, Functioning and Health (ICF) [4]. The ICF classification describes level of functioning (body functions, activities and participation) and disability (impairments, activity limitations and participation restrictions), hereby including the influence of contextual factors (environmental and personal) [5]. Although the ICF framework is based on a biopsychosocial paradigm, it has been criticised for the dominant position that disease takes within the framework and scheme, and alternative schemes have been proposed [6]. When asking patients with slowly progressive NMD about topics important to their quality of life, results show that not all topics are mentioned or emphasized by clinicians using the ICF framework [7]. These findings demonstrate the importance of how well-being is conceptualized in rehabilitation. Supporting patients in realizing a certain level of functioning requires being able to identify and monitor factors and activities that contribute to their well-being. To emphasize this need for assessing what patients are able to do in their real-life environments in order to realize well-being, rather than merely observing their functional status, there is a growing interest in applying the capability approach to healthcare [8]. This manuscript describes the protocol of a study that will apply the capability approach in designing and evaluating rehabilitation care for patients with neuromuscular diseases (Fig 1).

**Fig 2:**
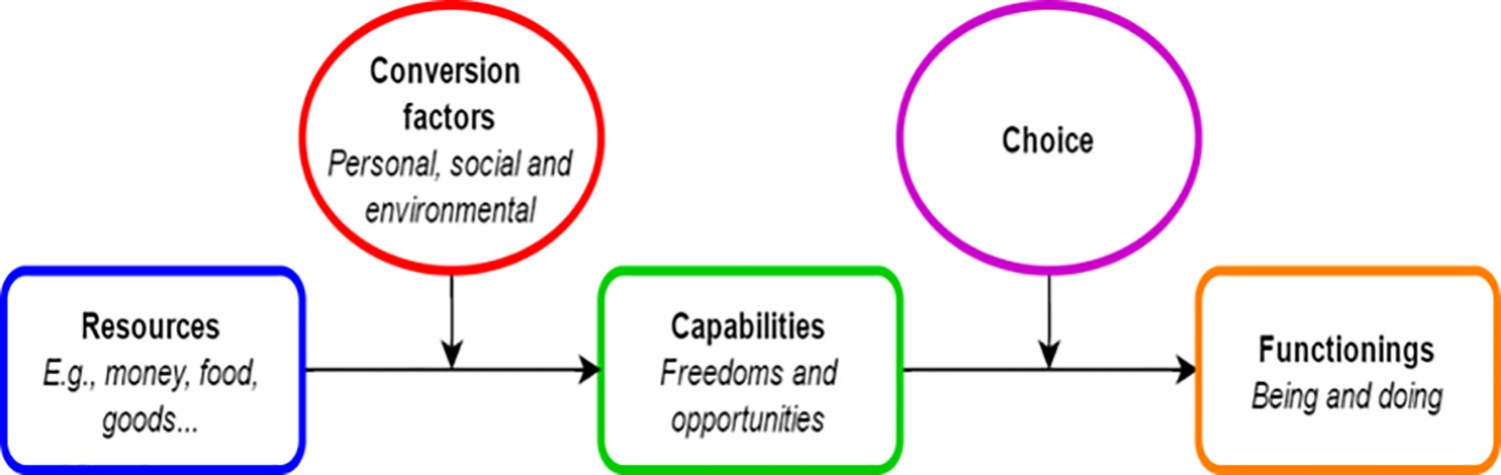
Schematic illustration of the theoretical framework of the capability approach. (adapted from Robeyns [9]).

The capability approach (Fig 2), developed by Nobel Prize Laureate Amartya Sen, offers a theoretical framework for studying well-being by analysing and evaluating an individual’s ability to achieve valuable *functionings* in life, that involves being able to be and do what is valued by people [9, 10]. Its central idea is that well-being is a function of a persons’ *capabilities*, the effective opportunities that someone has to achieve these valuable doings and beings. A person’s ‘capability set’ is the set of valuable options that he or she can choose from, whereas a person’s *functionings* are the realized options (e.g. working, resting, being literate, healthy, part of a community). According to the capability approach, a person’s capabilities are determined by the *resources* at one’s disposal, and personal and environmental characteristics (*conversion factors*) that allow someone to use available resources in such a way that it contributes to realizing valuable states of being. The capability approach implies that to promote well-being, we should expand a person’s capabilities. The foremost difference with other conceptualizations of well-being is that it focuses upon the freedoms and opportunities a person enjoys in living his or her life.

The capability approach provides a framework of well-being that revolves around people’s abilities to achieve certain ends given their resources and environmental factors. It could help in analysing well-being in a more integrated, personalized and realistic way. Integrating a capability perspective in clinical practice shifts attention from identifying the patient’s health problems and symptoms, towards exploring the patient’s actual opportunities in the light of their bodily impairments, and how this may contribute to their well-being. One of the strengths of the capability approach is its focus upon the reasoned choice and participation of individuals to identify the most valuable opportunities within their own context. Implementing the capability approach in care for patients with slowly progressive NMD might broaden the focus that is needed to fully provide person-centred care, potentially leading to a higher well-being for these patients.

Although the capability approach provides a broad theoretical framework to study and conceptualize well-being, more work is needed to be able to use it in clinical practice [11]. Thus far, the capability approach has been applied primarily in *evaluating* the impact of health-related states and interventions on well-being [8, 12]. Although a lot of effort has been invested in this important issue of how to use the capability approach in evaluation, there has not yet been a study that explicitly *designed* and *developed* a healthcare intervention based on the capability approach. Although it would be highly relevant to see whether such a use of the approach could help in enhancing the well-being of patients, applying this approach within healthcare interventions raises several challenges. Firstly, because the capability approach is a broad theoretical framework that consists of abstract terms that describe elements of well-being, its actual use requires several decisions with respect to its operationalization. In particular, to be able to use the approach, there is a need for explicitly selecting *which* capabilities are relevant to enhance and assess well-being in a particular context (i.e. what is the ‘capability set’ that you want to enlarge?). Secondly, applying the approach in delivering care raises the question whether healthcare professionals are responsible for protecting and enhancing the capabilities of patients. This would imply that healthcare professionals would support patients in aspects of their lives that are relevant to their capabilities (well-being), even when these are not (held to be) directly related to their disease state.

To address the challenges in applying the capability approach, we aim to develop *capability care* by making explicit choices and making use of quantitative and qualitative methods to carefully observe their implications for the delivery and impact of care. This study is the first example of such an attempt to design and deliver a healthcare intervention based on the capability approach. We will explore how rehabilitation care for patients with chronic progressive NMD can be based on this approach, developing an intervention that aims to improve patients’ well-being. The results of this study will contribute to our understanding of the factors involved in improving the care for patients with NMD, and expands our knowledge on how the capability approach can be applied in delivering and evaluating healthcare.

## Materials and methods

### Aim of the study

The primary objective of this study is to assess the impact of capability care on the well-being of patients with FSHD and DM1 in comparison to usual outpatient rehabilitation care. In addition, this study also tries to answer questions on how capability care can be designed and implemented and how its impact is evaluated.

### Study design

#### Patient groups

**Fig 3:**
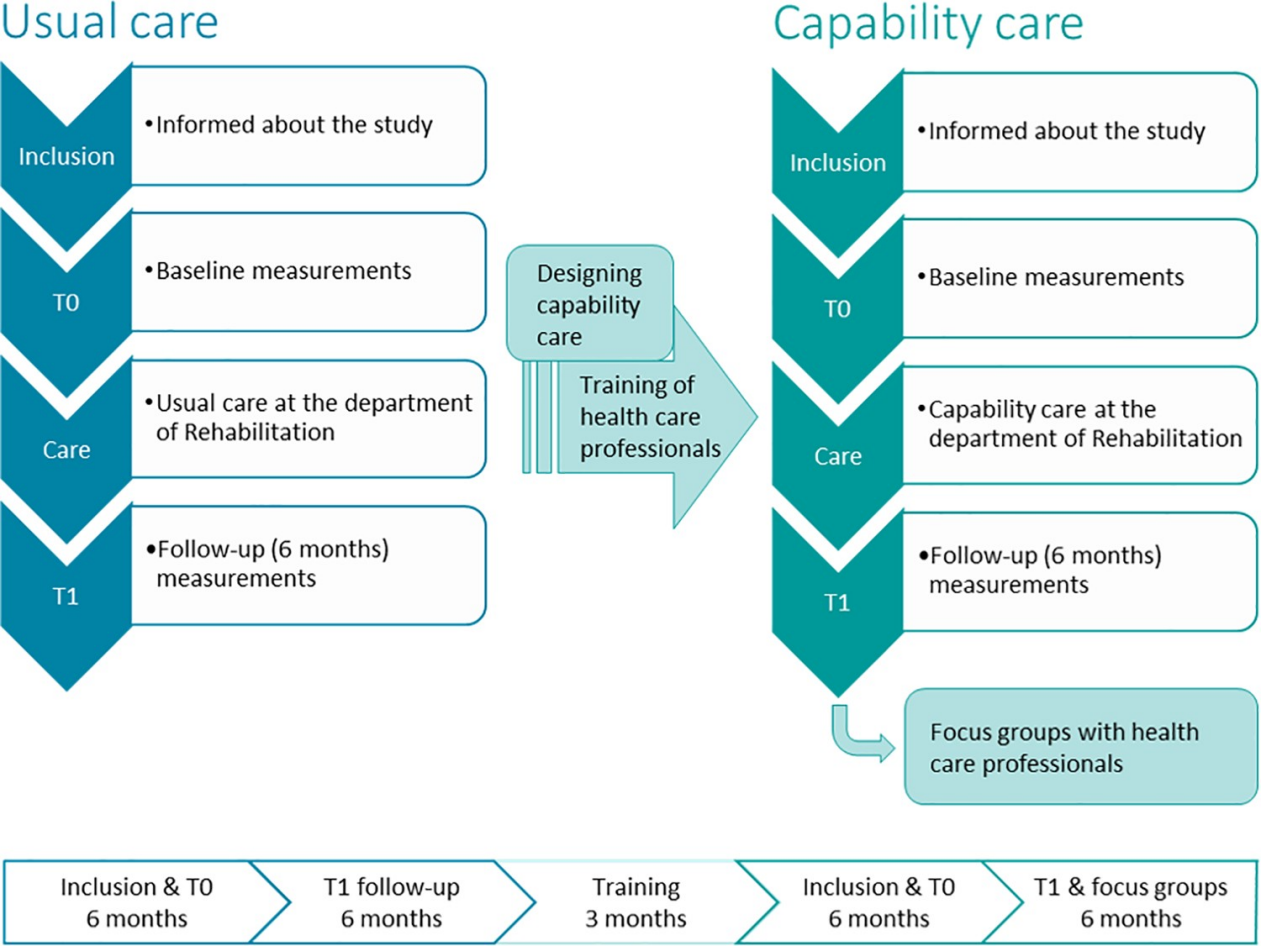
Flowchart of the ReCap-NMD study. Patients are included and baseline measurements take place before their first appointments at the department of Rehabilitation of the of the Radboudumc Center of Expertise for neuromuscular disorders. Follow-up measurements will take place after 6 months. In total, a period of one year per group is planned for inclusion and follow-up. In between usual care and capability care, a period of 3 months is planned to train the healthcare professionals in providing capability care.

Two patient groups are included in a controlled before and after design, and the impact of care on their well-being is assessed by using a mixed methods approach. The first group (n = 30) will be included during a period of 6 months, and they will receive usual multidisciplinary outpatient rehabilitation care. The observations made during usual care will be used to inform the development of capability care (as described below). Then, during a period of 3 months, members of the multidisciplinary outpatient rehabilitation care team of the Radboudumc Center of Expertise for neuromuscular disorders will be trained in providing capability care. Subsequently, the second group (n = 30) will be included during a period of 6 months, and they will receive multidisciplinary outpatient rehabilitation capability care. Both groups are followed up for a period of 6 months after inclusion. A schematic illustration of the study design can be seen in the flowchart below (Fig 3). Only patients are blinded with respect to the assignment into the usual care or capability care group.

#### Mixed methods approach

**Fig 4:**
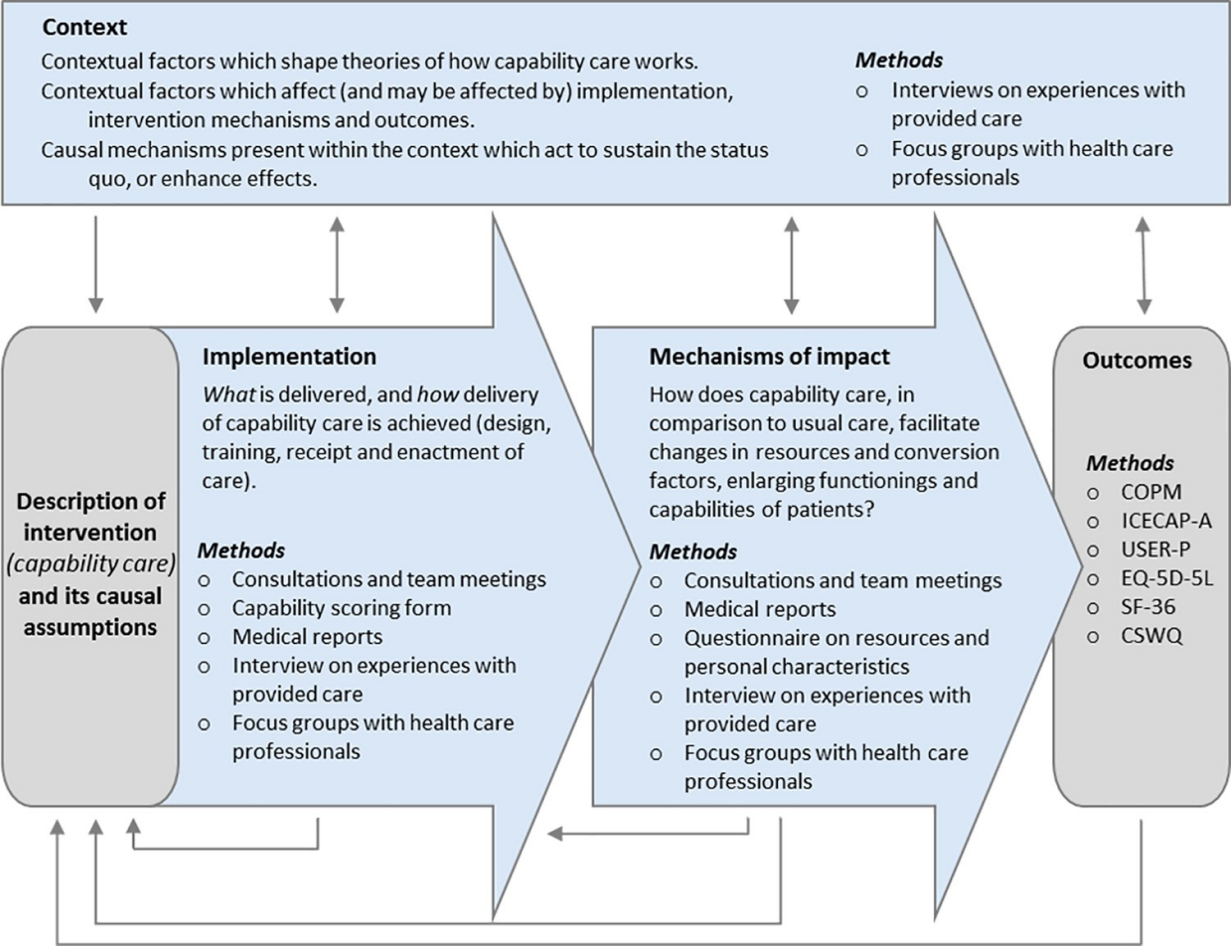
Process evaluation (adapted from Medical Research Council framework for process evaluation [13]). Blue boxes represent components of process evaluation, which are informed by the causal assumptions of the intervention, and inform the interpretation of (quantitative) outcomes.

COPM: Canadian Occupational Performance Measure; ICECAP-A: ICEpop CAPability measure for Adults; USER-P: Utrecht Scale for Evaluation of Rehabilitation Participation; EQ-5D-5L: EuroQol-5D-5L; SF-36: Medical Outcome Study Short Form-36; CSWQ: Capability Set for Work Questionnaire.

We make use of a mixed methods approach in which quantitative and qualitative data collection and analyses are combined to perform a process evaluation that provides complementary information to the quantitative outcomes [13]. The qualitative analyses can provide in-depth understanding of how the capability care should be, and was actually implemented, the mechanisms that explain its effects, and how the context affects implementation and impact. This process evaluation assists in interpreting the outcomes of the quantitative analyses, to be able to interpret the observed effects. In Fig 4, which is based on the MRC process evaluation framework [13], it is shown how the process evaluation relates to the understanding of the outcomes. To be able to assess the impact of care, and to monitor mechanisms and contextual factors, a diversity of qualitative and quantitative methods of data collection is used.

#### Measurements

Baseline measurements (T0) will take place 1–2 weeks before the first appointments with the healthcare professionals at the department of Rehabilitation of the Radboudumc Center of Expertise for neuromuscular disorders. Questionnaires will be completed online (or on paper if the patient prefers this) and the Canadian Occupational Performance Measure (COPM) interview will be performed by independent occupational therapy research assistants. Follow-up measurements (T1) will take place 6 months after the baseline measurements. Questionnaires will be completed online (or on paper if the patient prefers this), COPM re-scoring will be performed by the independent occupational therapy research assistants, and the interviews on experiences with provided care will be performed by researchers and research assistants. Patients in the usual care group and capability care group will undergo the same measurements. If assessments are not completed, the reasons will be documented and registered as missing data. Adverse events or irregularities affecting protocol adherence are registered by the primary researchers (BB and EP). Full ethical approval has been granted by the medical ethical reviewing committee CMO Regio Arnhem-Nijmegen (NL72794.091.20). The ReCap-NMD study has been registered at trialregister.nl (NL8946).

### Setting

Patients are recruited at the Radboudumc Center of Expertise for neuromuscular disorders, Nijmegen, The Netherlands, via either the department of Rehabilitation or the department of Neurology. The Radboudumc Center of Expertise for neuromuscular disorders is an (inter)national centre of expertise for FSHD and DM1. Patients are recruited and included before their appointments for ‘analysis and advice’ at the outpatient rehabilitation clinic of the Radboudumc Center of Expertise for neuromuscular disorders. Patients are nationally referred for diagnosis, analysis and advice. Both new referrals and patients having their annual check-up appointments at the Radboudumc Center of Expertise for neuromuscular disorders are eligible for inclusion in the current study.

### Participants

Eligible participants (based on a pre-screening performed by their physician or nurse practitioner) will receive study information and will be asked whether the research team is allowed to contact them by phone. After the initial phone call from the researcher (BB or EP) and receiving more detailed study information on paper, all eligible participants will be given at least 7 days before they are contacted by the researcher again to consider their response and to answer any questions that the patient might have. If the patient is interested in participating, informed consent will be obtained before inclusion. After obtaining informed consent, inclusion criteria will be verified by an independent research assistant before conducting the COPM interview.

Table 1 provides an overview of the applied inclusion and exclusion criteria. All patients should be at least 18 years of age and have a confirmed and genetically proven diagnosis (by a neurologist) of FSHD or DM1. Patients should also have a current rehabilitation aim, defined as at least 3 important activities for which they would like to improve their performance, irrespective of whether they have been treated previously. Additionally, patients should be in a mentally stable condition and have sufficient mastery of the Dutch language.

**Table 1:** Inclusion and exclusion criteria of the ReCap-NMD study.

Inclusion criteria	Exclusion criteria
1) confirmed genetically proven diagnosis of FSHD or DM1 by neurologist	1) active or previously major psychotic, psychiatric or depression episodes
2) 18 years or older	2) acquired brain injury (e.g. stroke, traumatic brain injury)
3) a current rehabilitation aim	
4) in a mentally stable condition	3) severe cognitive problems (e.g. severe dementia) in which case the rehabilitation treatment is affected and/or patients are not able to fill out the questionnaires
5) sufficient mastery of the Dutch language to participate in conversation with the healthcare providers and research assistant and to fill in questionnaires	
	4) limited life expectancy (e.g. due to cancer)

### Intervention

#### Usual care

Usual care at the department of Rehabilitation of the Radboudumc Center of Expertise for neuromuscular disorders for patients diagnosed with FSHD or DM1 consists of a multidisciplinary outpatient ‘analysis and advice’ trajectory. It includes consultations with the multidisciplinary outpatient rehabilitation team: a rehabilitation physician (or physician assistant), nurse practitioner, and allied healthcare professionals such as a physical therapist, occupational therapist, speech and language therapist and dietician. Which appointments are scheduled is dependent on a pre-screening by either the rehabilitation physician or nurse practitioner.

Patients are invited for a day of outpatient consultations with the selected healthcare professionals. On this day the patient first meets the healthcare professionals in individual consultations; followed by a multidisciplinary meeting in which the healthcare professionals discuss their findings; and lastly the patient meets with the rehabilitation physician again to discuss the advice from the team. Depending on the patient’s needs, this advice usually consists of one of the following: 1) additional outpatient consultations at the department of Rehabilitation of the Radboudumc; 2) referral to a NMD rehabilitation care team of a regional rehabilitation clinic within the Netherlands; 3) referral to primary allied healthcare (e.g. physical therapy, occupational therapy) close to the patient’s home; or 4) no further treatment necessary. The final decision on further treatment is the result of a shared decision-making process between the patient and rehabilitation physician, based on the team’s advice. If considered necessary, the rehabilitation physician will be in touch after 3 months for follow-up.

#### Capability care

**Fig 5:**
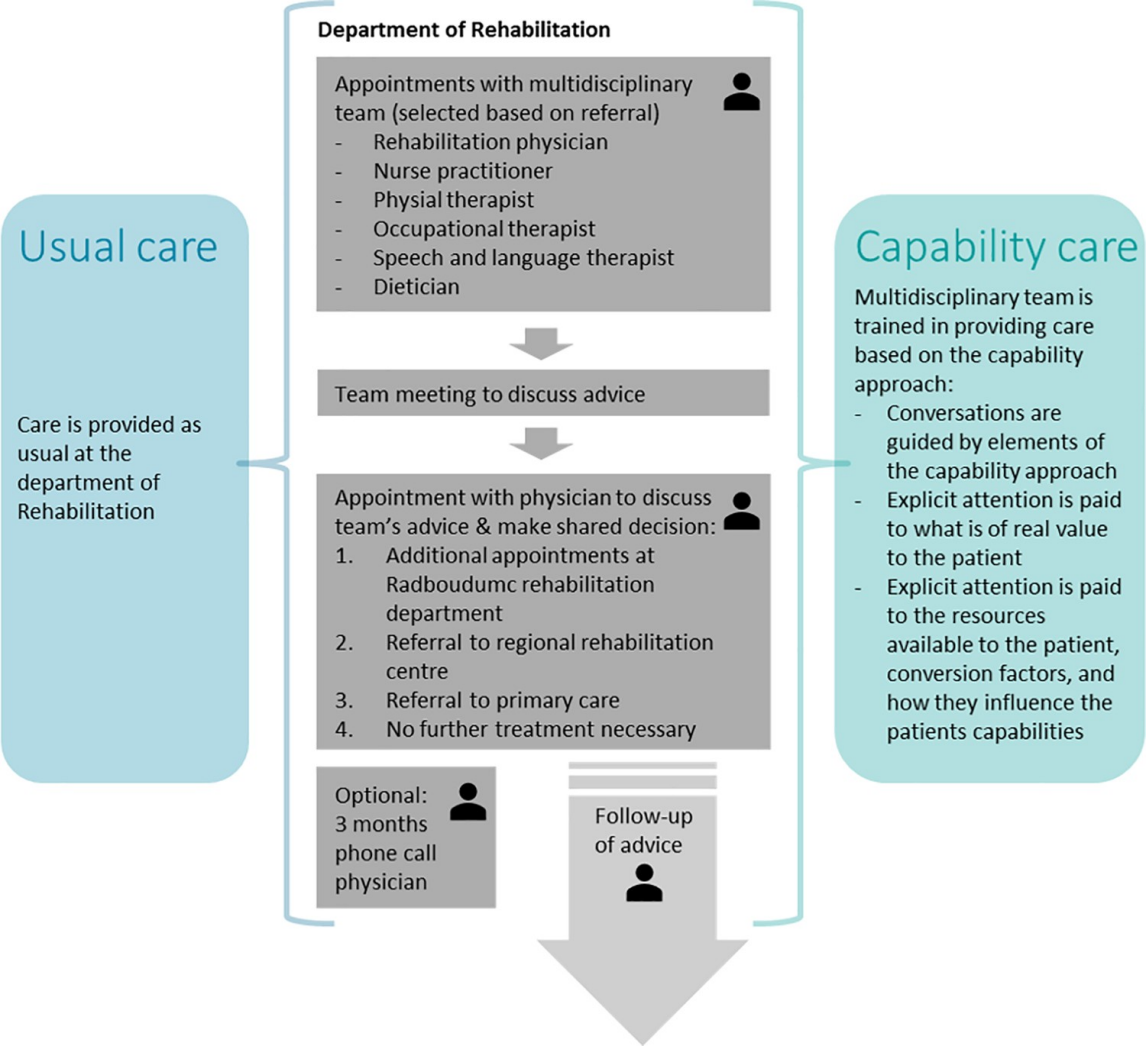
Flowchart of usual care and capability care pathway for the patient. The patient is visualised with the icon of a person, which shows the patient journey where the patient is present during the consultations. The patient is not present during the team meeting.

To develop capability care, a combination of theoretical exploration, discussions within the project group, conversations with other experts from the capability approach domain and rehabilitation field, and data collected from the usual care group will be used. The data that will be analysed qualitatively is obtained from retrospective interviews with participating patients, audio recordings of the outpatient consultations with healthcare professionals and the multidisciplinary team meetings, and the medical records of the patients. Based on that, we will identify the capabilities that are relevant to the well-being of NMD patients, the factors that are important in providing capability care, and provide a clear description of how these factors will be implemented in rehabilitation care. However, no changes will be made in the care process itself; i.e. in the capability care pathway patients will receive the same number of outpatient consultations as in the usual care pathway (Fig 5).

To implement capability care, a period of three months is planned to train all involved healthcare professionals (i.e. the same professionals as in usual care) in providing capability care (Fig 3). As capability care needs to be developed, the exact training methods will depend on what capability care will look like. Our expectation is that capability care will be provided by changing the focus of the consultations, which requires knowledge about the theoretical background of the capability approach, how this applies to patients with NMD, and conversation techniques on how to guide a consultation based on the capability approach. Possible methods to train the team consist of combination of developing an information guide or manual, presentations on the capability approach and its application in healthcare, practising with conversation techniques, and developing material to facilitate the conversations.

### Outcome measures

The primary objective of this study is to assess the impact of capability care on the well-being of patients with NMD. To be able to do this, a set of quantitative outcome measures is used to evaluate and compare different impacts of rehabilitation care on patients with NMD. Additionally, qualitative data is used to perform a process evaluation, providing insight in contextual factors, mechanisms of impact, and a deeper understanding of the impact of care on the daily life of patients.

The primary outcome is the difference in the COPM performance and satisfaction scores between both groups (corrected for the value at baseline) [14]. The COPM is a semi-structured interview designed to help patients identify daily occupations that are meaningful to them and difficult for them to perform. Patients are asked to prioritize 3 to 5 important occupations they would like to improve, for which a score is given for performance from 1 (impossible) to 10 (very well possible) and for satisfaction with performance from 1 (not satisfied) to 10 (extremely satisfied). The COPM is a reliable and valid instrument [15–17], and has been shown to be a sensitive and clinically relevant outcome measure in a number of rehabilitation studies [15, 18–22]. The COPM results are also noted in the patient file, visible for the healthcare team (so that the occupational therapist does not need to repeat the COPM, as this is often used in clinical practice).

Secondary measures include the ICEpop CAPability measure for Adults (ICECAP-A) [23], Utrecht Scale for Evaluation of Rehabilitation Participation (USER-P) [24], EuroQol-5D (EQ-5D-5L) [25, 26], Medical Outcome Study Short Form-36 (SF-36) [27] and the Capability Set for Work Questionnaire (CSWQ) [28]. This broad range of measures provides a way to uncover the diverse impact of care, and to compare the validity of different instruments.

The qualitative data collection includes a (self-developed) questionnaire on resources and personal characteristics, interviews with participating patients and their partners, audio-recordings of consultations between patients and healthcare professionals, audio-recordings of healthcare professionals’ team meetings, medical records, and focus groups with healthcare professionals after both groups have completed their treatment. The data from the usual care group will be used to develop the capability care intervention (as described above); the data from both groups and the focus groups with healthcare professionals will be used to perform a process evaluation (as described above, Fig 4).

Table 2 provides an overview of all data collection methods.

**Table 2:** Methods of data collection.

Participant	Outcome	Data collection method	Baseline (T0)	6 months (T1)	Random selection	End of follow-up
**Patient**	**Primary**					
	Performance on and satisfaction with meaningful daily occupations	Canadian Occupational Performance Measure (COPM)–performance and satisfaction score (16)	x	x		
**Patient**	**Secondary**					
	Capabilities	ICEpop CAPability measure for Adults (ICECAP-A) (25)	x	x		
	Participation	Utrecht Scale for Evaluation of Rehabilitation Participation (USER-P) (26)	x	x		
	Health-related quality of life	EuroQol-5D (EQ-5D-5L) (27, 28)	x	x		
	Health-related quality of life	Medical Outcome Study Short Form-36 (SF-36) (29)	x	x		
	Work capabilities	Capability Set for Work Questionnaire (CSWQ) (30)	x	x		
	**Qualitative data**					
**Patient**	‘Resources’ and ‘Conversion factors’ (capability approach)	Questionnaire on resources and personal characteristics (self-developed)	x	x		
	Delivery of care in terms of capability approach	Interview on experiences with provided care (audio-recorded)		x		
**Partner (if possible)**	Delivery of care in terms of capability approach	Interview on experiences with provided care (audio-recorded)		x		
**Other**	Delivery of care in terms of capability approach	Audio recordings of consultations with the healthcare professionals			x	
	Delivery of care in terms of capability approach	Audio recordings of multidisciplinary team meetings (audio-recorded)			x	
	Delivery of care in terms of capability approach	Medical records			x	
	Delivery of care in terms of capability approach	Focus groups with healthcare professionals on their experience with providing capability care (audio-recorded)				x

### Sample size calculation

The primary outcome measure is the COPM, which consists of the performance score (COPM-P) and satisfaction score (COPM-S). Both COPM-P and COPM-S scores at 6 months after baseline (T1), corrected for the value at baseline (T0), will be used. We consider the intervention as successful only if for both outcomes the capability care group performs significantly better than the usual care group.

The expected difference between both groups is 1.4 for COPM-P and 1.9 for COPM-S. These values represent a clinically relevant improvement as perceived by the client [19]. The standard deviations in both groups and for both outcomes are expected to be 1.9 at time T1 [20]. Based on these assumptions, a sample size of at least 24 patients per group will yield a power of 80% to show a statistical difference between the intervention and the usual care group at a significance level of 0.05. These calculations are based on ANCOVA-models with the outcome at 6 months as dependent variables, corrected for the value at baseline and an assumed correlation of 0.5 between the outcome values at T0 and T1 [29]. Considering dropout, we decided to include 30 participants in each group.

### Statistical analysis

#### Primary and secondary outcome measures

The primary outcome measure will be analysed using an ANCOVA model with the follow up scores (T1) as dependent variable. Covariates will be the T0 scores, age, gender and diagnosis. For an intervention-effect to be present, the scores on both the COPM-P and COPM-S will need to show a significant change. A p-value below 0.05 will be considered significant.

Similar analyses (ANCOVA model) will be performed for these secondary outcome measures: ICECAP-A preference-weighted index score, ICECAP-A non-weighted sum score, USER-P, CSWQ total score, EQ-5D-5L preference-weighted index score, EQ-5D-5L non-weighted sum score, SF-36 preference-weighted index score, SF-36 non-weighted sum score. The CSWQ overall question will be analysed using a Mann-Whitney U test on the difference score between T0 and T1. A p-value below 0.05 will be considered significant.

To compare the capability measure (ICECAP-A) with the health-related quality of life measures (EQ-5D-5L, SF-36), multiple contingency tables will be created to compare the number of patients that show significant changes in their well-being based on the ICECAP-A, EQ-5D-5L and SF-36 ANCOVA models. These comparisons will be performed both for the sum scores and the preference-weighted scores (i.e. based on utility estimates derived from members of the general population). An analysis solely based on the preference-weighted scores may conclude that the measure is not responsive, when in fact, it could be that the descriptive system of the measure is responsive but the change is not valued [30].

#### Qualitative data

Results from qualitative analyses will be used for [1] developing the capability care intervention, [2] the assessment of the implementation process, [3] exploring how capability care facilitates changes, and [4] exploring the influence of context (parts of process evaluation; see Fig 4 for an overview). These qualitative analyses will be conducted as described below.

#### Analysis of qualitative data

For the interviews with patients and partners, a constant comparative method [31] will be applied using the following steps:

1. 3 to 5 patients and, if possible, partners will be interviewed.
2. Interviews will be fully audio recorded, and intelligent (non-verbatim) transcripts will be created. The transcripts will capture the meaning of what is being said, and focus on parts of the interview that help answering the research questions related to how rehabilitation and capability care influence the well-being of patients.
3. The transcripts will be analysed using a deductive qualitative content analysis [32] approach in which the researcher starts with predetermined categories and coding rules from an existing theory. In our study this theory is the capability approach, with resources, conversion factors, functionings, and capabilities as its central categories. The coding will be done using the software ATLAS.ti 8 Windows.
4. The researchers will discuss the coding, to ensure consistency in interpretation, and discuss whether new topics need to be addressed in the next interviews.
5. Another 3–5 interviews are being held and transcribed, coded and discussed.
6. The final 5–10 interviews are being held, transcribed and coded and evaluated whether these confirm the preliminary findings or whether new insights arise. New patients and partners are interviewed, transcribed and coded until no new insights arise (data saturation has been reached).
7. The findings are discussed with the whole research group and overarching themes may be identified. These themes are formulated and illustrated with original quotes.

For the analysis of the at verbatim transcripts of the focus groups, a random selection of the at verbatim transcriptions of the consultations and team meetings, and a random selection of the medical records, the following steps will be used:

1. Codes will be allocated to meaningful text parts answering the research questions and using a deductive qualitative content analysis approach [32]. This coding will be done using the software ATLAS.ti 8 Windows.
2. The researchers will discuss codes and categories.
3. The codes and categories are discussed with the whole research group and overarching themes may be identified.
4. Themes and subthemes are formulated and illustrated with original quotes.

Additionally, to evaluate whether capability care is genuinely applied, and to compare the differences between usual and capability care, a scoring form for delivery of capability care will be developed. This scoring form will be used to score a random selection of the audio recordings on the occurrence of treatment elements that are based on the capability approach. The scoring will be performed independently by two blinded research assistants.

The credibility of the findings is supported by triangulation of the findings from the interviews (patients and partners), consultations, multidisciplinary team meetings, medical records and questionnaires on resources and personal characteristics, and focus groups.

## Discussion

The ReCap-NMD study is the first example of an attempt to implement capability care, and will contribute to an understanding of how to design and evaluate a capability application in healthcare. Because the capability approach provides a broad theoretical framework, designing such a study is challenging and several choices need to be made in operationalizing the approach. Firstly, we will explain and discuss our choices regarding the measurement of the impact of capability care on well-being, specifically our choice of using the COPM as our primary outcome measure. Then, we will explain our choice for a before-after study design and how this helps us in developing capability care. Thirdly, we will address our choice to include patients with two different forms of slowly progressive NMD (FSHD and DM1).

To explain our choices concerning the measurement of well-being, it is important to discuss the nature of well-being. The concept of well-being refers to evaluative judgments about major aspects of a life situation or life path [33]. It is not a single determinate thing, or any number of things, lying out there to be measured. When measuring well-being, specific decisions need to be made in order to define what is valuable (e.g. what constitutes well-being), how the realization of valuable aspects of life can be observed, what the purpose is of the evaluation (e.g. enhance our understanding, guide public policy), the standpoint from which the evaluation can be undertaken, and which particular conception of well-being guides the evaluation [33]. Therefore, there exists a plurality of conceptualizations and operationalizations (constructs, measures) of well-being [34]. This plurality is a reflection of the contextual nature of well-being itself [34]. What constitutes well-being depends on the particular situation, and can differ between persons. To acknowledge this, we make use of a mixed-methods approach that allows us to measure the impact of care on the well-being of patients in both quantitative and qualitative terms. In addition, we have selected both personalised outcome measures (COPM), generic HRQoL measures (EQ-5D and SF-36), and measures inspired by the capability approach (ICECAP-A), to compare different operationalizations of well-being and their influence on evaluating the effectiveness of care.

We have chosen the COPM as the primary outcome measure as it is an operationalisation of well-being that matches the particular context of the current study. The COPM is a personalised outcome measure which focuses on the ability to perform self-identified meaningful occupations and the satisfaction in performing these occupations (well-being). The COPM has been developed in the occupational therapy field and has been used in rehabilitation practice and studies. It is a reliable and valid [15–18] outcome measure that captures a relevant aspect of well-being for patients: the performance and satisfaction with daily occupations that are regarded important by the patient. The patients prioritize three to five daily occupations that they would like to change or improve prior to their rehabilitation treatment. Moreover, it can reasonably be expected that rehabilitation care will have an effect on the performance of and satisfaction with these daily occupations that are important for the patient to improve or change, as a number of rehabilitation studies have shown [15, 16, 18–22]. This makes the COPM an appropriate measure for observing changes during rehabilitation care. In addition, daily occupations that are regarded important by the patient can be compared to functionings in the capability model which are regarded to be the realized beings and doings deemed valuable by a person. Our hypothesis is that providing rehabilitation care based on the capability approach will help patients and professionals in identifying relevant daily occupations and ways of realizing them, which could lead to higher performance and satisfaction scores.

As secondary outcome measures, we have chosen a selection of established participation (USER-P), health-related quality of life (SF-36, EQ-5D-5L) and capability outcome measures (ICECAP-A, CSWQ), to be able to obtain a broader assessment of the well-being of patients. The USER-P is a rehabilitation outcome measure that focuses on the domain of participation. The ICECAP-A and CSWQ are outcome measures developed based on the capability approach, where the ICECAP-A measures well-being in general, and the CSWQ is focused on capabilities and well-being in work. The SF-36 and EQ-5D-5L are measures of health-related quality of life (HRQoL). The SF-36 and EQ-5D have been used by HRQoL studies in NMD but its use needs further investigation [35, 36]. Especially in the context of chronic and progressive diseases, the sensitivity of SF-36 and EQ-5D to subtle changes in the condition of patients during rehabilitation is limited. Comparing the SF-36 and EQ-5D with capability-based outcome measures could provide more information on the relevance of using these instruments to assess well-being in NMD and their sensitivity to changes in the well-being of patients.

Next to using quantitative measures, we will also perform a process analysis using qualitative data. This provides us with information on what is actually happening during rehabilitation care (audio recordings of consultations with healthcare professionals and team meetings; medical records, how the patients and their partners have experienced the care (retrospective interviews with patients and if possible their partners), and how the healthcare professionals have experienced the difference between usual care and capability care (focus groups with healthcare professionals). This will help us in understanding the underlying mechanisms of usual and capability care. Comparing the quantitative outcome measures and triangulating these with the qualitative results will provide us with more insight into the effects of capability care and its critical elements.

With respect to challenges related to developing capability care, we have chosen to use a controlled before-after design. We have both pragmatic and methodological reasons for choosing this approach. Because the healthcare team providing specialized care to NMD patients at Radboudumc is too small to divide into two groups (providing usual or capability care) it is not possible to randomly assign the patients to a treatment group (as in a randomized controlled trial). And due to the nature of capability care training, it is also not possible for a professional trained in capability care to provide capability care to one patient and usual care to the other. Professionals will be trained to look at the patient from a different perspective, and after being trained it is not realistic to argue that the professional can let go of this perspective. Besides these pragmatic reasons, the before-after design allows us to learn from the usual care group to be able to develop capability care. An analysis of the qualitative data of usual care can serve as input for discussions with other experts from the capability approach domain and the rehabilitation field on which capabilities are relevant to the well-being of NMD patients and how they could be realized in capability care.

The current study includes both patients with FSHD and DM1, which involves a high heterogeneity between participants. Although this could increase the complexity of the analyses, we have several reasons for including patients with both types of NMD. The Radboudumc is a (inter)national centre of expertise for both diseases and can provide excellent care for these patients. Both diseases are chronic degenerative NMD with a slowly progressive character, and disease course and symptoms not only show a high variability between NMD but also within patient groups with the same NMD diagnosis. Despite this variation, an important commonality between both diseases is that patients struggle to maintain a level of functioning that leads to a fulfilling life. This matches the goal of rehabilitation treatment to support patients in finding a way to achieve and maintain this level of functioning, leading to a higher well-being. In addition, rehabilitation treatment is always individualized and depends on the patient’s situation, wishes and aims. Therefore, although the treatment is adjusted to personal differences, we do not expect a difference in the comparative effectiveness of usual care and capability care between both diseases. However, to be certain, we decided to analyse our data using diagnosis as a covariate to take the possibility of differences into account.

## Study status

The study is ongoing. Inclusion of participants in the usual care group is completed. The inclusion of participants in the capability care group will start on February 1, 2022, and is expected to be completed before August 1, 2022.

## Supporting information

S1 Checklist (DOCX)

S1 File (PDF)
