# Supplementary material for: Care for capabilities: Implementing the capability approach in rehabilitation of patients with neuromuscular diseases. Study protocol of the controlled before-after ReCap-NMD study
Source: PLoS One. 2021 Dec 21;16(12):e0261475. doi: 10.1371/journal.pone.0261475 (PMC8691629; doi:10.1371/journal.pone.0261475)
Supplement: S1 File — (PDF) [file pone.0261475.s002.pdf]

RESEARCH PROTOCOL

Rehabilitation & Capability Care in NMD (ReCap-NMD)

NL72794.091.20

PROTOCOL TITLE 'Capability care: what matters most to patients with neuromuscular diseases?'

**Protocol ID**	**109580**
**Short title**	**Rehabilitation & Capability Care in NMD (ReCap-NMD)**
**Version**	**1.2**
**Date**	**01-09-2020**
**Project leader**	Dr. E.H.C. Cup<br>Senior researcher, department of Rehabilitation, Radboudumc<br>Route 932<br>Postbus 9101<br>6500 HB Nijmegen<br>024-3614893
**Principal investigators**	B. Bloemen MSc<br>PhD candidate, department for Health Evidence, Radboudumc<br>Route 133<br>Postbus 9101<br>6500 HB Nijmegen<br>024-3655356<br><br>E.J. Pijpers MSc<br>PhD candidate, department of Rehabilitation, Radboudumc<br>Route 932<br>Postbus 9101<br>6500 HB Nijmegen<br>024-3655231
**Sponsor**	Radboudumc, department of Rehabilitation<br>Route 932<br>Postbus 9101<br>6500 HB Nijmegen
**Subsidising party**	Prinses Beatrix Spierfonds
**Independent expert**	Dr. H.J.R. van Duijnhoven<br>Rehabilitation physician, department of Rehabilitation, Radboudumc<br>Route 932<br>Postbus 9101<br>6500 HB Nijmegen<br>024-3614892

PROTOCOL SIGNATURE SHEET

Name	Signature	Date
**Head of Department:**<br>Prof. Dr. A.C.H. Geurts<br>Head of Department of Rehabilitation, Radboudumc		**01-09-2020**
**Project leader:**<br>Dr. E.H.C. Cup<br>Senior researcher / Head of Occupational Therapy department, Radboudumc		**01-09-2020**

TABLE OF CONTENTS

1. INTRODUCTION AND RATIONALE 9
2. OBJECTIVES 12
3. STUDY DESIGN 13
4. STUDY POPULATION 14
4.1 Population (base) 14
4.2 Inclusion criteria 14
4.3 Exclusion criteria 14
4.4 Sample size calculation 15
5. TREATMENT OF SUBJECTS 16
5.1 Investigational treatment 16
5.1.1 Usual care 16
5.1.2 Capability care 16
5.2 Use of co-intervention 17
5.3 Escape medication 17
6. METHODS 18
6.1 Study parameters/endpoints 18
6.1.1 Main study parameter/endpoint 19
6.1.2 Secondary study parameters/endpoints 19
6.1.3 Other study parameters 21
6.2 Randomisation, blinding and treatment allocation 23
6.3 Study procedures 24
6.4 Withdrawal of individual subjects 25
6.4.1 Specific criteria for withdrawal 25
6.5 Replacement of individual subjects after withdrawal 25
6.6 Follow-up of subjects withdrawn from treatment 26
6.7 Premature termination of the study 26
7. SAFETY REPORTING 27
7.1 Temporary halt for reasons of subject safety 27
7.2 AEs, SAEs and SUSARs 27
7.2.1 Adverse events (AEs) 27
7.2.2 Serious adverse events (SAEs) 27
7.2.1 Suspected unexpected serious adverse reactions (SUSARs) 27
7.3 Follow-up of adverse events 28
8. STATISTICAL ANALYSIS 29
8.1 Primary study parameters 33
8.2 Secondary study parameters 33
8.3 Other study parameters 34
8.3.1 Developing capability care 34
8.3.2 Assessment of treatment fidelity 35
8.3.3 Exploring how capability care facilitates changes in resources, conversion factors, functionings and capabilities 36

8.3.4 Exploring how the context influences the implementation, mechanisms and outcomes of capability care 37
8.4 Interim analysis 37
9. ETHICAL CONSIDERATIONS 38
9.1 Regulation statement 38
9.2 Recruitment and consent 38
9.3 Benefits and risks assessment, group relatedness 38
9.4 Compensation for injury 39
9.5 Incentives 39
10. ADMINISTRATIVE ASPECTS, MONITORING AND PUBLICATION 40
10.1 Handling and storage of data and documents 40
10.2 Monitoring and Quality Assurance 40
10.3 Amendments 41
10.4 Annual progress report 41
10.5 Temporary halt and (prematurely) end of study report 41
10.6 Public disclosure and publication policy 41
11. REFERENCES 42

LIST OF ABBREVIATIONS AND RELEVANT DEFINITIONS

AE	Adverse Event
ANOVA	Analysis of Variance
CA	Capability Approach
CCMO	Central Committee on Research Involving Human Subjects; in Dutch: Centrale Commissie Mensgebonden Onderzoek
COPM	Canadian Occupational Performance Measure
CSWQ	Capability Set for Work Questionnaire
DM1	Myotonic Dystrophy type 1
EQ-5D-5L	EuroQol-5D-5L
FSHD	Facioscapulohumeral dystrophy
HTA	Health Technology Assessment
IC	Informed Consent
ICECAP-A	ICEpop CAPability measure for Adults
ICF	International Classification of Functioning Disability and Health
METC	Medical research ethics committee (MREC); in Dutch: medisch-ethische toetsingscommissie (METC)
NMD	Neuromuscular disease(s)
RCT	Randomized Controlled Trial
(S)AE	(Serious) Adverse Event
SF-36	Medical Outcome Study Short-Form-36
Sponsor	The sponsor is the party that commissions the organisation or performance of the research, for example a pharmaceutical company, academic hospital, scientific organisation or investigator. A party that provides funding for a study but does not commission it is not regarded as the sponsor, but referred to as a subsidising party.
SPSS	Statistical Package for the Social Sciences
SUSAR	Suspected Unexpected Serious Adverse Reaction
USER-P	Utrecht Scale for Evaluation of Rehabilitation Participation
WMO	Medical Research Involving Human Subjects Act; in Dutch: Wet Medisch-wetenschappelijk Onderzoek met Mensen

SUMMARY

Rationale: High quality care of patients with inheritable neuromuscular diseases (NMD) requires a multi-faceted personalized approach. The capability approach provides a new conceptualization of wellbeing focusing on a) the real opportunities that patients have to be and do things they have reason to value and b) the dynamic interaction between access to resources (e.g., medication, aids), personal characteristics, physical and social environment, and such opportunities. In this study we want to investigate whether providing care based on the capability approach (capability care) has an added value in the rehabilitation of patients with NMD.
Our research question: What is the effect of capability care on the quality of life of patients with NMD in comparison with usual care?
Hypothesis: The quality of life of adult patients with NMD can be enhanced by providing capability care in comparison to usual care.

Objective:
Primary Objective:
To compare the quality of life of patients with NMD receiving capability care versus usual care, by:

- using the Canadian Occupational Performance Measure (COPM) to compare current performance on, and satisfaction with, activities in the domains of self-care, productivity and leisure that are meaningful to the patient.

Secondary Objectives:

- To develop rehabilitation care for patients with NMD that is based on the capability approach.
- To assess treatment fidelity in designing capability care, training of the health care professionals in providing capability care, delivery of capability care, and receipt and enactment of capability care by the patients with NMD.
- To determine how capability care, in comparison to usual care, facilitates changes in resources and conversion factors, enlarging functionings and capabilities.
- To compare the capabilities outcome of patients with NMD receiving capability care versus usual care, using the ICECAP-A that covers five attributes (e.g. attachment, stability, achievement, enjoyment, autonomy) of wellbeing.
- To compare the participation outcome (USER-P) of patients with NMD receiving capability care versus usual care.
- To compare the work capabilities outcome (CSWQ) of patients with NMD receiving capability care versus usual care.
- To determine whether the use of a broader measure of quality of life (ICECAP-A), compared to a health-related quality of life measure (EQ-5D-5L, SF-36), leads to other outcomes in terms of assessing the benefits of care.

Study design: This study will include two groups of thirty adult patients with NMD. The first group will be provided with optimal care as usual, and followed up for a period of 6 months. Then, during a period of 3 months, members of the multidisciplinary outpatient rehabilitation

care team at Radboudumc will be trained in providing capability care. Subsequently, the second group will be provided with capability care, and followed up for a period of 6 months.

Study population: Patients are included when a neurologist has confirmed the neuromuscular diagnosis Facioscapulohumeral muscular dystrophy (FSHD) or myotonic dystrophy type 1 (DM1). Participants are 18 years or older and in a mentally stable condition. Patients need to have a current rehabilitation aim to be included.

Intervention: One group of patients will receive usual rehabilitation care. The other group of patients will receive care based on the capability approach (capability care). Health care professionals will be trained to apply the capability approach in consultation and care to identify a) capabilities of patients and b) barriers or promoting factors of patients' capabilities. The health professionals learn how to support the patient in thinking about capabilities. It is expected that one of the main changes is that the consultations will help patients in reflection on what really matters to them, how their chronic illness interferes with achieving desired ends, and how rehabilitation can help the patient to address this.

Main study parameters/endpoints:
Primary outcome:
Difference in the Canadian Occupational Performance Measure (COPM) between both groups (corrected for the value at baseline).
Secondary outcomes:
Difference between both groups in the the ICEpop CAPability measure for Adults (ICECAP-A), Utrecht Scale for Evaluation of Rehabilitation Participation (USER-P), the EuroQol-5D-5L (EQ-5D-5L), the Medical Outcome Study Short-Form-36 (SF-36) and the Capability Set for Work Questionnaire (CSWQ) score.
Other study parameters:
Audio recordings of consultations with the healthcare professionals and the multidisciplinary team meetings, medical reports, questionnaire on resources and personal characteristics, interviews with patients and partners, focus groups with healthcare professionals.

Nature and extent of the burden and risks associated with participation, benefit and group relatedness: The participants in the intervention group receive healthcare based on the capability approach (capability care). It is expected that this type of healthcare is more person-centred and will lead to a higher quality of life for patients with neuromuscular disease. The provision of rehabilitation care with an explicit focus on strengthening patients' capabilities is not expected to incur greater risk than treatment as usual.
Participants in both the usual care and capability care groups undergo two measurement moments. Measurement moments contain an interview (1 hour) and self-report questionnaires (electronically or on paper) (50 minutes). The interview at baseline will take place over the phone or via video-call, the interview after 6 months will take place at the Radboudumc or at a location convenient for the participant (at home) so that travel time is limited. The measurements are non-invasive and no health risks are expected. Participants possibly are confronted with their limitations by the questionnaires or during the interviews. The research assistants are trained occupational therapists who can address concerns of participants or advise participants to visit other health professionals.

1. INTRODUCTION AND RATIONALE

From a focus on disease to a broader focus on wellbeing

Facioscapulohumeral muscular dystrophy (FSHD) and myotonic dystrophy type 1 (DM1) are relatively frequently occurring inheritable neuromuscular diseases (NMD), with a prevalence of 12/100.000 for FSHD and 10/100.000 for DM1 (Deenen et al., 2014; van Engelen, 2015). Since there is no curative treatment available, and disease course and severity is highly variable, high quality care for these patients requires a multi-faceted personalized approach, flexible to their diverse clinical expression, that focuses on regaining and maintaining a level of functioning that enables them to lead a satisfying life. To support this, both neurological and rehabilitation care have adopted the WHO International Classification of Disability, Functioning and Health (ICF) to describe and evaluate health states in neuromuscular diseases (Bos, Kuks, & Wynia, 2015). Introduction of this ICF model has shifted the focus in clinical practice to a more holistic and integrative classification of 'health and disability' with a focus on the patient's bodily functions and structures, activities and participation. However, the ICF does not have an explicit and broad focus on wellbeing that may be needed to fully support person-centred care that is sensitive to all domains of patients' lives that are affected by these diseases.

In addition to the development of more integrated and personalised approaches to care, measures of subjective wellbeing are needed, complementary to objective capacity measures such as standardized exercise tests, imaging and genetic counselling, to adequately monitor and evaluate the impact of interventions. Currently, wellbeing of patients with DM1 and FSHD is primarily measured by using generic health-related quality of life (HRQoL) instruments such as the Medical Outcome Study Short Form-36 (SF-36) (Landfeldt et al., 2019; Winter et al., 2010). Because the validity of the SF-36 as a measure of HRQoL in DM1 warrants further investigation, and using preference-based instruments (i.e. linked to utilities) may provide additional and complementary information about the impact of disease, the exploration of a broader range of measures to assess wellbeing is needed to adequately uncover the diverse effects of interventions (Landfeldt et al., 2019). This may help in assessing the additional benefits and costs, besides health gains, of interventions that aim to enhance quality of life.

Conceptualizing wellbeing: the capability approach

The capability approach (CA), developed by Nobel Prize Laureate Amartya Sen, offers a theoretical and normative framework for studying wellbeing by analysing and evaluating an individual's ability to achieve valuable functionings in life (Robeyns, 2005; Sen, 1993). The CA states that wellbeing should be understood in terms of *capabilities*, or the real freedom that people have to be and do things they have reasons to value. Consequently, the protection and promotion of wellbeing demands an expansion of peoples' actual opportunities. According to the CA, these are a result of a dynamic interaction between access to *resources*; personal characteristics, physical and social environment (*conversion factors*); and such opportunities (*functionings*), see also Figure 1.

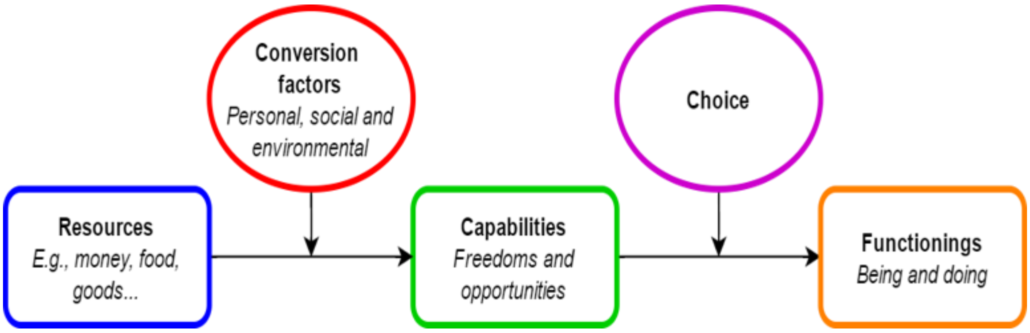

Figure 1. Schematic illustration of the theoretical framework of the capability approach (adapted from Robeyns (Robeyns, 2005)). Its central idea is that wellbeing is a function of a persons' capabilities, the freedom that someone experiences to be and do what they have chosen to aspire. These opportunities are determined by the resources at ones disposal, and personal and environmental characteristics that allows someone to use available resources in a way that contributes to realizing valuable states of being.

As an example, the use of a wheelchair can be analysed in terms of capabilities. A wheelchair becomes a 'valuable' resource when a disabled person uses it to realize personal values, such as visiting friends, going to church or enjoying nature (Mitra, 2017). However, two disabled persons may differ in their ability to convert the use of a wheelchair to realizing desirable activities due to differences in personal, social and environmental characteristics and context. Conversion factors are those (distinguishing) factors which limit or promote the conversion of the wheelchair as a resource into capabilities and functionings (Robeyns, 2017). These factors may be the patient's social skills, physical abilities, gender, but also psychological characteristics such as autonomy, competency, self-stigma and self-efficacy; social conversion factors could be support from significant others or the attitude or stigma towards people with disabilities in the neighbourhood; environmental conversion factors could be the absence of safe roads and elevators in public spaces for proper wheelchair use.

One of the strengths of the CA framework is the respect for human diversity within groups, communities or populations. The starting point of the approach is the reasoned choice and participation of individuals to identify the most valuable opportunities within their own context. Therefore, integrating a capability perspective in clinical practice shifts attention from identifying the patient's health problems and symptoms, due to disability and incapacities, towards exploring the patient's actual opportunities in the light of their bodily impairments, and how this may contribute to a higher quality of life.

When applied in the context of clinical care, the capability approach entails that health care providers explore together with patients and their relatives what really matters to them, what actions are needed for making this happen, and agree on who is going to pursue those actions, where and when. This quest is conducted collaboratively, while not ignoring the reality of the limitations or special needs that may be associated with NMD. Drawing on the capability approach, this quest will focus on accessibility of requisite resources, presence of necessary conversion factors and functionings, and their interdependencies (Sen, 1992).

<u>Capability applications in healthcare</u>

The CA provides a broad theoretical framework to study and conceptualize wellbeing, but more work is needed to use it in developing actual applications (Robeyns, 2006). The CA

has already been applied in healthcare to assess people's engagement in physical activity, patient empowerment, assessing multidimensional poverty in a health setting, and in evaluating the impact of care interventions for different population groups (Mitchell, Roberts, Barton, & Coast, 2017). For example, Van Engelen, Van der Wilt and Meerman have performed two pilot studies: a) a capability impact assessment of patients with myotonic dystrophy type 1 participating in the experimental (person-centred cognitive behavioural therapy and aerobic exercise) and control group of the OPTIMISTIC trial, and b) the identification of capabilities in children with FSHD on the basis of participatory observations and interviews to identify the conditions which facilitate or limit wellbeing for these children (in collaboration with dr. C. Erasmus). Preliminary results of 10 interviews with participants of the OPTIMISTIC trial indicate that patients in the experimental group experience more positive changes in different capability domains, especially: life, practical reasonableness and 'work and play' in comparison to usual care.

A recent study has shown the value of the capability approach in assessing the impact of healthcare services and subsequent rehabilitation by applying the approach in evaluating the capabilities of children with cochlear implants (Rijke et al., 2019). In this study it was observed that children with cochlear implants who perform well on standard clinical tests (e.g. linguistic and auditory tests), still experienced serious limitations in their desired functioning. These findings suggest that a capability approach may reveal aspects of what is achieved through rehabilitation that might otherwise remain unnoticed, contributing to finding ways to improve the patients wellbeing.

Contributions of this study

All current applications of the CA primarily concern the description and evaluation of health-related states of being, and the assessment of the impact of social and care interventions. A common rationale behind all these studies is the assumption that the CA, and instruments based on this approach (e.g. questionnaires, survey), more appropriately captures the impact of interventions in terms that are considered valuable by patients and decision makers. Although a lot of effort has been invested in showing and elaborating this, there has not yet been a study that explicitly used the CA in developing and implementing an intervention prospectively.

To the best of our knowledge, this study is the first example of an inquiry into designing and delivering a healthcare intervention for people with chronic (progressive) diseases based on the CA. This study will explore how rehabilitation care for patients with NMD can be based on the CA, developing an intervention that aims to improve patients' wellbeing. The results of this study will contribute to our understanding of the factors involved in improving the care for patients with NMD, and expands our knowledge on how the capability approach can be applied in delivering and evaluating healthcare.

2. OBJECTIVES

Primary Objective:
To compare the quality of life of patients with NMD receiving capability care versus usual care, by:

- using the Canadian Occupational Performance Measure (COPM) to compare current performance on, and satisfaction with, activities in the domains of self-care, productivity and leisure that are meaningful to the patient.

Secondary Objectives:

- To develop rehabilitation care for patients with NMD that is based on the capability approach.
- To assess treatment fidelity in designing capability care, training of the health care professionals in providing capability care, delivery of capability care, and receipt and enactment of capability care by the patients with NMD.
- To determine how capability care, in comparison to usual care, facilitates changes in resources and conversion factors, enlarging functionings and capabilities.
- To compare the capabilities outcome of patients with NMD receiving capability care versus usual care, using the ICECAP-A that covers five attributes (e.g. attachment, stability, achievement, enjoyment, autonomy) of wellbeing.
- To compare the participation outcome (USER-P) of patients with NMD receiving capability care versus usual care.
- To compare the work capabilities outcome (CSWQ) of patients with NMD receiving capability care versus usual care.
- To determine whether the use of a broader measure of quality of life (ICECAP-A), compared to a health-related quality of life measure (EQ-5D-5L, SF-36), leads to other outcomes in terms of assessing the benefits of care.

3. STUDY DESIGN

Two patient groups are included in a controlled before and after design. The first group of 30 patients will be included during a period of 6 months; they will be provided with optimal care as usual, and followed up for a period of 6 months after inclusion. The data from this group will be used to develop and design the intervention (capability care), as described in more detail in chapter 5.1. Then, during a period of 3 months, members of the multidisciplinary outpatient rehabilitation care team at Radboudumc will be trained in providing capability care. Subsequently, the second group of 30 patients will be included during a period of 6 months; they will be provided with capability care, and followed up for a period of 6 months after inclusion.

The baseline measurements will take place before the appointments with the health care professionals at the department of rehabilitation. The baseline measurements include an interview for the COPM (by an independent research assistant) and a number of questionnaires that patients can digitally fill out from home (online) without supervision.

The follow-up measurements will take place 6 months after the baseline measurements. Patients will be asked to fill out the same questionnaires as in the baseline measurement. Additionally, an interview will be conducted in which the COPM is re-scored and patients (and if possible their partners) are asked on how they experienced the healthcare provision. COPM re-scoring will be done by an independent research assistant; interviews will be conducted by both the researchers and research assistants.

Patients in both the usual care group and capability care group will undergo the same measurements. All study assessments not completed will be documented as missing data.

The study design is illustrated in the flowchart below (fig. 2).

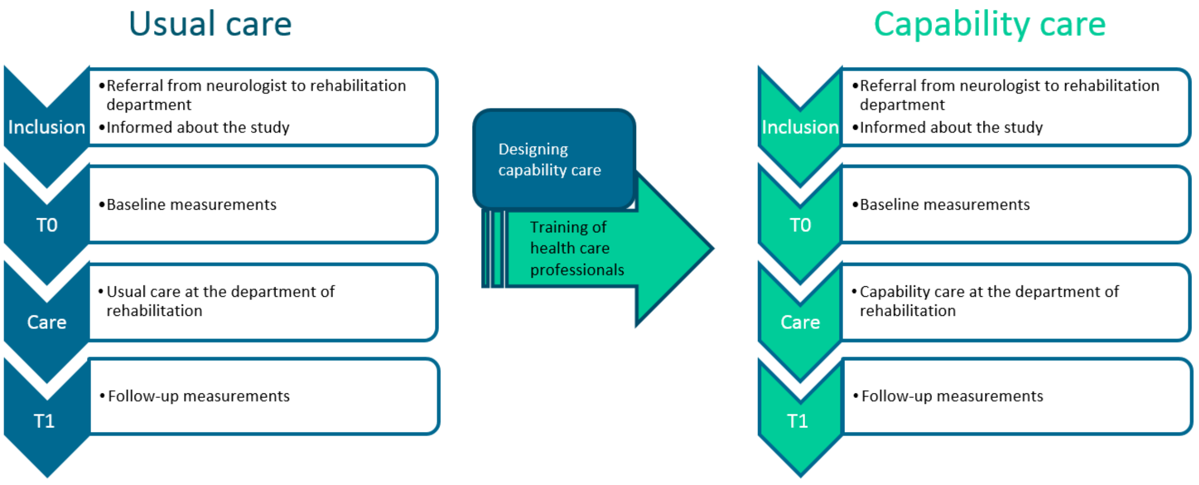

Figure 2. Flowchart of the study design.

4. STUDY POPULATION

4.1 Population (base)

The diseases addressed in this project, facioscapulohumeral dystrophy (FSHD) and myotonic dystrophy type 1 (DM1), are relatively frequently occurring neuromuscular diseases. DM1 is a chronic inheritable disease with a multi-systemic nature. It is the most common type of muscular dystrophy in adults (Harper, 2001). Clinical characteristics are muscle weakness and generalized distal (and later on) proximal weakness of muscles, ptosis, and myotonia (disturbed relaxation of muscles), which lead to severe fatigue, substantial physical functional impairment, and restricted social participation (Okkersen et al., 2018). FSHD is characterized by slowly progressive asymmetrical weakness of the facial and shoulder girdle muscles, followed by leg and trunk muscles (Mul et al., 2016; Padberg, 1982). In DM1 and FSHD, disease course and severity are highly variable, even within families, ranging from asymptomatic gene carriers to wheelchair bound patients. The prevalence of DM1 is estimated to be > 10/100.000 people according to OPTIMISTIC (van Engelen, 2015). The prevalence of FSHD is estimated at 12/100.000 (Deenen et al., 2014).

This study will include 60 adult patients with DM1 (n=30) and FSHD (n=30) referred to the Radboudumc muscle centre, national expertise centre for both FSHD and DM1. Eligible participants will receive study information from their neurologist or rehabilitation physician and will be asked whether the research team is allowed to contact them over the phone. All participants will be given at least 7 days after receiving the study information before they are contacted by the research team again to consider their response and to answer any questions that the patient might have. If the patient is interested in participating, informed consent will be obtained before inclusion. We will obtain informed consent from all participants taking part in this study (see section 9.2). Inclusion criteria will be checked by the research assistant before the COPM interview. We do not expect a large number of exclusions after obtaining informed consent, as the treating physician has screened participants before contacting them with study information.

4.2 Inclusion criteria

In order to be eligible to participate in this study, a subject must meet all of the following criteria:

- confirmed diagnosis of FSHD or DM1 by neurologist
- 18 years or older
- a current rehabilitation aim
- in a mentally stable condition
- sufficient mastery of the Dutch language to participate in conversation with the health care providers and research assistant and to fill in questionnaires
- informed consent (written)

Patients that have been treated previously and have new rehabilitation aims can be included.

4.3 Exclusion criteria

A potential subject who meets any of the following criteria will be excluded from participation in this study:

- patients that have one of the following comorbidities:
    - active or previously major psychotic, psychiatric or depression episodes

- acquired brain injury (e.g. stroke, traumatic brain injury)
- severe cognitive problems (e.g. severe dementia) in which case the rehabilitation treatment is affected and/or patients are not able to fill out the questionnaires
- a limited life expectancy (e.g. due to cancer)

4.4 Sample size calculation

In this study the primary outcome measure is the COPM, which consists of the performance score (COPM-P) and satisfaction score (COPM-S). Both COPM-P and COPM-S scores at 6 months after baseline (T1), corrected for the value at baseline (T0), will be used. Both outcomes are considered equally important and the researchers consider the proposed treatment as successful only if for both outcomes the capability care group performs significantly better than the group that is given treatment as usual.

For COPM-P a difference of 1.4 is expected between the usual care group and the capability care group at time T1 (when scored blinded). For COPM-S this expected value is 1.9. These values represent a clinically relevant improvement as perceived by the client (Eyssen et al., 2011). The standard deviations in both groups and for both outcomes are expected to be 1.9 at time T1 (Veenhuizen et al., 2019).

Treatment is considered to be successful only if for both outcomes a difference can be shown. That means that a sample size calculation has to be done for the two outcomes combined in which the correlation between the outcomes should be taken into account. This correlation will be positive in this study; it is expected to be close to 1. The most conservative sample size (highest sample size) will be obtained if we require a type I error of at most the significance level for either test (corresponds to a correlation close to one) and the multiplication of the powers of the two tests is equal to the desired power of the total test (corresponds with independent test-statistics). For this situation, and under the assumptions given in the previous paragraph, a sample size of at least 24 patients per group will yield a power of 80% to show a statistical difference between the treatment and the usual-care groups at significance level 0.05. These calculations are based on ANCOVA-models with the outcome at 6 months as dependent variables, corrected for the value at baseline and an assumed correlation of 0.5 between the outcome values at T0 and the T1 (Borm, Fransen, & Lemmens, 2007).

In reality, the correlation between the outcomes is expected to be close to 1 (Eyssen et al., 2011). In that case the type I error, as well as the power, of the combined test is close to the type I error and power of a single test. For the outcome COPM-P (the outcome with the smallest expected effect), a sample size of at least 23 is necessary to show a difference between the two groups based on an ANCOVA-model corrected for the baseline value (with assumed correlation between the outcome values at T0 and T1 equal to 0.5) at level 0.05 with power 0.80.

Based on the power calculations, we would need to include 23 or 24 (worst case scenario) participants in each group. Considering dropout, we decided to include 30 participants in each group. Additionally, this will make it more feasible to triangulate results of the secondary outcomes with the primary outcome.

5. TREATMENT OF SUBJECTS

5.1 Investigational treatment

5.1.1 Usual care

Usual care at the department of Rehabilitation of the Radboud University Medical Center, for patients diagnosed with FSHD or DM1, starts with a diagnosis or follow-up appointment with a neurologist and a referral to the department of rehabilitation medicine. At the department of rehabilitation medicine the patient will receive consultations with a multidisciplinary healthcare team. Depending on the referral, the healthcare team consists of a rehabilitation physician, physician assistant, nurse, physiotherapist, occupational therapist, speech-language therapist, and/or dietician. Patients are invited for a day of appointments with these healthcare professionals. On this day the patient first meets the healthcare professionals in individual consultations; then the healthcare team has a meeting to discuss their advice; and lastly the patient meets with the rehabilitation physician or physician assistant again to discuss the advice from the team. Depending on the patient's needs, this advice usually consists of one of the following: 1) additional appointments at the department of Rehabilitation of the Radboud University Medical Center; 2) referral to a regional rehabilitation centre within the Netherlands; 3) referral to primary health care (e.g. physiotherapy, occupational therapy) close to the patient's home. In all cases, the rehabilitation physician will be in touch after 3 months for follow-up. In figure 3 this pathway is presented in a flowchart.

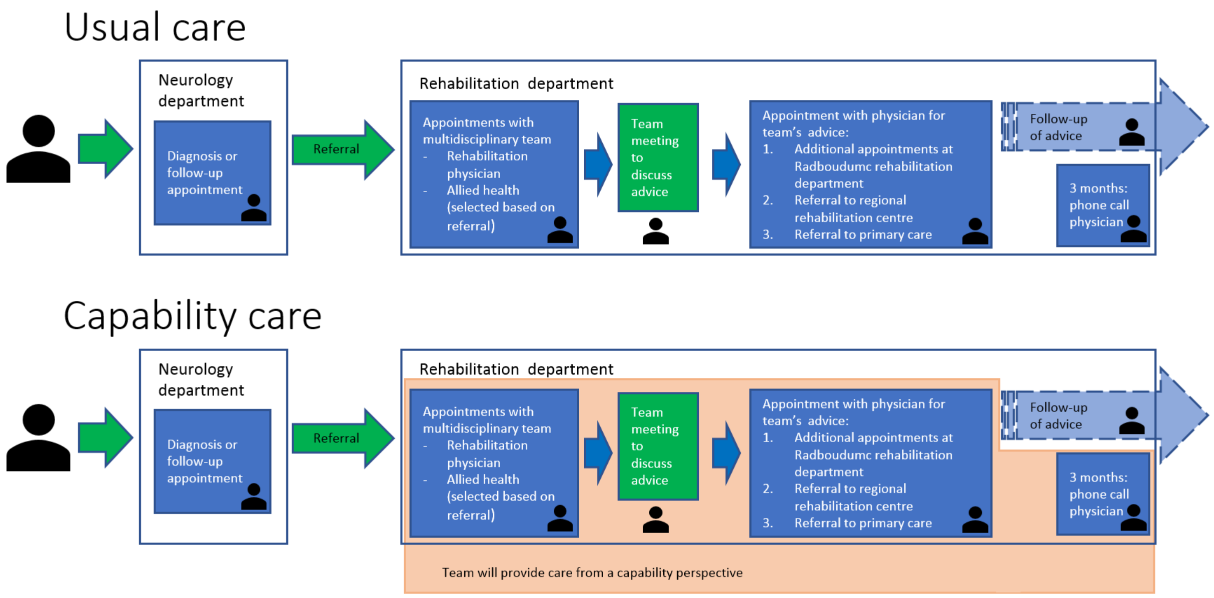

Figure 3. Flowchart of usual care and capability care pathway for the patient.

5.1.2 Capability care

Development of capability care

As recommended by the Medical Research Council (Moore et al., 2014, 2015), there needs to be a clear description of the intervention and its causal assumptions before implementing the intervention. To develop the capability application in healthcare ('capability care') for patients with neuromuscular diseases, we will use a combination of theoretical exploration,

discussions within the project group and with other experts from the capability approach domain and the rehabilitation field, and using insight obtained by analysing the data collected from the usual care group.

After 6 months, the usual care group will be followed up, where one of the measures is a retrospective interview. This interview focuses on how the patients have experienced the healthcare and how this has changed several aspects in their lives, and on the limitations and barriers that prevent making these changes (see interview guide in appendix F; outcome measures). These interviews will be qualitatively analysed in terms of the capability approach. Additionally, the consultations with the health care professionals (audio-recorded), the multidisciplinary team meetings (audio-recorded) and the medical reports from the patient files will be analysed qualitatively in terms of the capability approach. This step is needed to determine in what respect usual care can be improved when looking through the capability lens. The results of this qualitative analysis will be discussed within the project team ,with the health care providers and with experts from the capability approach field, to determine the changes that need to be made to provide capability care (development of the capability care intervention) and provide a clear description of the intervention and its causal assumptions.

In the capability care pathway patients will receive the same number of consultations and performance tests, but the CA framework will be used to guide conversations and this may lead to other advice during these conversations and a different overall advice from the multidisciplinary rehabilitation team. The health care providers will learn to identify a) capabilities and b) barriers or promoting factors, and to support the patient to think in terms of capabilities in order to realize activities that are of value to him or her. Analysing resources, conversion factors and functionings, alternatives for reaching these goals will be specified. It is expected that one of the main changes is that the consultations will help patients in reflection on what really matters to them, how their chronic illness interferes with achieving desired ends, and how the rehabilitation can help the patient to address this.

Implementation of capability care
A period of three months is planned to train all health care professionals in providing capability care. As capability care needs to be developed, the exact training methods will also need to be determined based on what capability care will look like. Our expectation is that capability care is provided by changing the focus of the consultations, which requires knowledge about the theoretical background of the capability approach, how this applies to patients with NMD, and conversation techniques on how to guide a consultation based on the CA. Possible methods to train the team consist of combination of developing an information guide or manual, presentations on the capability approach and its application in healthcare, practising with conversation techniques, and developing material to facilitate the conversations.

5.2 Use of co-intervention

Not applicable.

5.3 Escape medication

Not applicable.

6. METHODS

6.1 Study parameters/endpoints

The primary objective of this study is to assess the impact of capability care on the quality of life of NMD patients. To be able to do this, a diverse set of quantitative outcome measures is used to evaluate, and compare, different outcome effects of rehabilitation care for patients with NMD.

In addition to evaluating impact, this study also tries to answer questions on how capability care can be designed and how its impact is realized. These questions are important because there are limitations to what evaluation using quantitative outcome measures can achieve, and we want to learn how capability care should be developed and implemented. Therefore, we make use of a mixed methods approach in which quantitative and qualitative analyses are combined to perform a process evaluation that provides complementary information to qualitative outcomes (Moore et al., 2014, 2015). The qualitative analyses can provide in-depth understanding of how the capability care should, and was actually, implemented, the mechanisms that explain its effects, and how the context affects implementation. This process evaluation assists in interpreting the outcome results of the quantitative analyses, to be able to draw conclusions on why the observed effects were seen. In figure 4, which is based on the MRC process evaluation framework, it is shown how the process evaluation relates to the analysis of outcomes. The idea behind this framework is that the effects of an intervention depend on the way in which the intervention is implemented, its mechanisms of action, and contextual factors. Qualitative methods, as described in 6.1.3, will provide information on these elements.

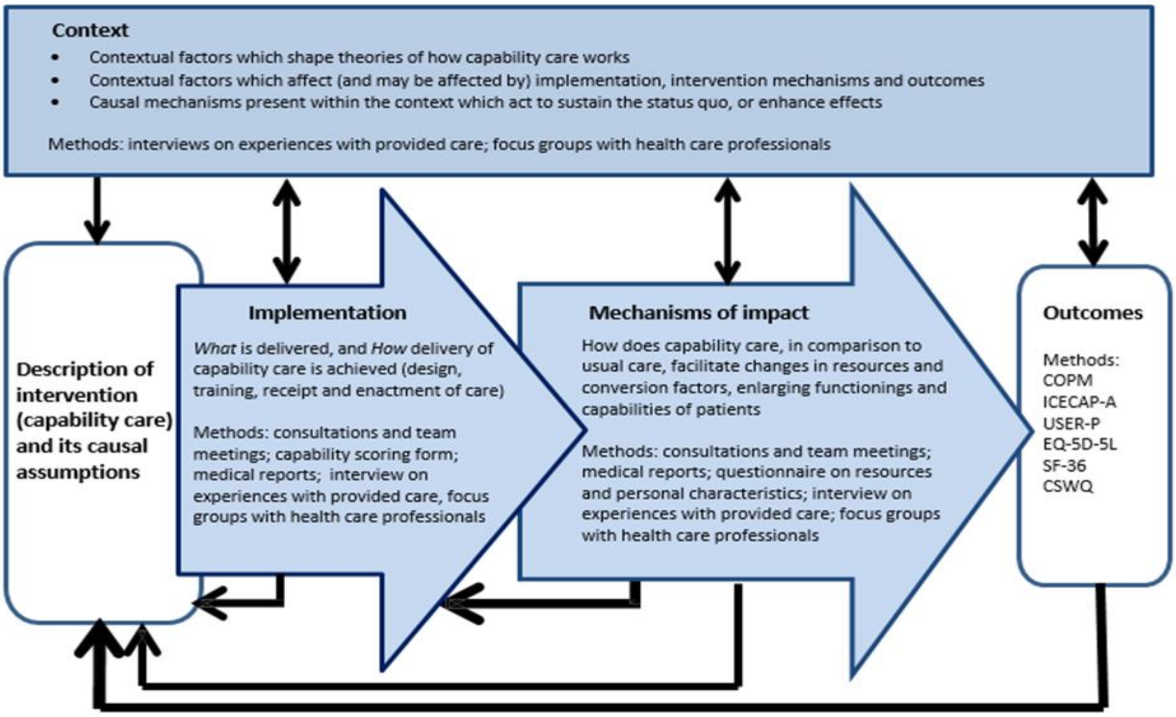

Figure 4. Process evaluation (adapted from Medical Research Council framework for process evaluations (Moore et al., 2014)). Blue boxes represent components of process evaluation, which are

informed by the causal assumptions of the intervention, and inform the interpretation of (quantitative) outcomes.

6.1.1 Main study parameter/endpoint

The main study parameter is the difference in the Canadian Occupational Performance Measure (COPM) between both groups (corrected for the value at baseline).

Canadian Occupational Performance Measure (COPM)

The COPM asks patients to identify daily activities which they want to do, need to do or are expected to do by encouraging them to think about a typical day in their life. Then they are asked to identify which of these activities are difficult for them to do. It covers areas such as personal care, functional mobility, community management, (un)paid work, household activities, play / school, hobbies, sports, and socialization (Law et al., 1998). Of the activities that are identified as difficult to perform, patients are asked to score the importance from 1 (not important) to 10 (extremely important). After having scored importance, patients are asked to prioritize 3 to 5 important daily occupations. For these occupations a score is given for performance from 1 (impossible) to 10 (very well possible) and for satisfaction with performance from 1 (not satisfied) to 10 (extremely satisfied). The mean scores for performance (COPM-P) and for satisfaction (COPM-S) will be used as endpoints. The follow-up scoring will be performed blinded (the patient is blinded to their own baseline scores). The optimal cut-off value of the COPM for evaluating improvement perceived by the client is 1.35 for the mean performance score (COPM-P) and 1.90 for the mean satisfaction score (COPM-S) when scored blinded (Eyssen et al., 2011).

6.1.2 Secondary study parameters/endpoints

The secondary study parameters are:

- Difference between both groups in the ICEpop CAPability measure for Adults (ICECAP-A), Utrecht Scale for Evaluation of Rehabilitation Participation (USER-P), the EuroQol-5D (EQ-5D-5L), the Medical Outcome Study Short-Form-36 (SF-36) score and the Capabilities Set for Work Questionnaire (CSWQ).

ICEpop CAPability measure for Adults (ICECAP-A)

The ICECAP-A is a measure of capability for the general adult (18+) population, focusing on wellbeing in a broader sense, rather than health (Al-Janabi, Flynn, & Coast, 2012). It comprises five attributes: attachment (ability to have love, friendship and support), stability (ability to feel settled and secured), achievement (ability to achieve and progress in life), enjoyment (ability to experience enjoyment and pleasure), and autonomy (ability to be independent). Each attribute corresponds to one item with four response levels. Depending on the item, the levels range from 'all' to 'not any' or 'a lot' to 'not any'. The patient is asked to indicate which level best describes their current situation, for each of the five items. These descriptions of different states of wellbeing can be converted to a single index value by using a standard value set. These standard value sets, or tariff scores, represent the average values attributed to different states within a given descriptive system (such as the ICECAP-A) by a representative sample of people (of a specific population). A total index value is obtained by summing these preference-based weights corresponding to levels of each

domain. The scale of this score ranges from 0 to 1, with 0 representing no capability and 1 representing full capability (Flynn et al., 2015).

<u>Utrecht Scale for Evaluation of Rehabilitation Participation (USER-P)</u>
The USER-P consists of 31 items and measures 3 aspects of participation: frequency of activities, experienced participation restrictions, and satisfaction with participation. The Frequency scale measures how much time is spent on vocational activity and leisure and social activity with a response scale ranging from 0=none at all to 5=36 h per week or more/ or 19 times per month or more. The Restriction scale measures experienced participation restrictions as a result of the health condition. Item scores range from 0=not possible to 3= without difficulty. A “not applicable” option is available for every item. The Satisfaction scale measures satisfaction with various aspects of participation. Item scores range from 0=very dissatisfied to 4=very satisfied. A “not applicable” option is available for the items on vocational activity and partnership relation. Sum scores are calculated per scale and converted to a 0-100 scale; higher scores indicate better levels of participation (higher frequency, less restrictions, higher satisfaction) (Post et al., 2012; van der Zee, Baars-Elsinga, Visser-Meily, & Post, 2013; van der Zee, Post, Brinkhof, & Wagenaar, 2014; van der Zee et al., 2010).

<u>EuroQol-5D (EQ-5D-5L)</u>
The 5-level EQ-5D was introduced by the EuroQol group (EuroQol Research Foundation, 2019; Herdman et al., 2011) to provide a simple, generic and sensitive measure of health-related quality of life for clinical and economic evaluations. The EQ-5D-5L essentially consists of 2 pages - the EQ-5D-5L descriptive system and the EQ visual analogue scale (EQ VAS). The EQ-5D-5L descriptive system comprises the following 5 dimensions: mobility, self-care, usual activities, pain/discomfort and anxiety/depression. Each dimension has 5 levels: no problems, slight problems, moderate problems, severe problems, and extreme problems. The respondent is asked to indicate his/her health state by ticking (or placing a cross) in the box against the most appropriate statement in each of the 5 dimensions. This decision results in a 1-digit number expressing the level selected for that dimension. The digits for 5 dimensions can be combined in a 5-digit number describing the respondent’s health state. Each health state can also be converted into a single index value using one of the standard EQ-5D-5L value sets which represent societal preference weights for the health state. These weights, sometimes referred to as ‘utilities’, are often used to compute Quality-Adjusted Life Years (QALYs) or use in health economic analyses. Health state index scores generally range from less than 0 (where 0 is the value of a health state equivalent to death; negative values represent states as worse than dead) to 1 (the value of full health). These health state preferences often represent national or regional values and can therefore differ between countries. Therefore, Dutch reference values will be used (Versteegh et al., 2016).

<u>Medical Outcome Study Short Form-36 (SF-36)</u>
The SF-36 is a health-related quality of life measure, consisting of 36 questions covering 8 domains: physical functioning (PF), role limitations due to physical health problems (RP), bodily pain (BP), general health perceptions (GH), vitality (VT), social functioning (SF), role limitations due to emotional problems (RE), and general mental health (MH) (Aaronson et al., 1998; Ware, Snow, Kosinski, & Gandek, 1993).
The SF-36 is scored in two steps. First, the response for each item is recoded with a value from 0-100. Second, an average value is calculated for the items in each of the eight scales.

Missing data are ignored and the scale score is calculated without the missing item. If more than 50% of the items are missing from any one scale, it cannot be calculated (Litwin, 2002). The SF-36 is sensitive to change in health-related quality of life over time and treatment. Higher scores indicate a higher health-related quality of life (Aaronson et al., 1998).
Based on a selection of SF-36 items, the SF-6D can be derived. The SF-6D is composed of six multi-level dimensions, and any patient who completes the SF-36 can be uniquely classified according to the SF-6D. The SF-6D provides a means for using the SF-36 in health economic evaluation by estimating a preference-based index value based on preference weights obtained from a sample of the general population. Because no Dutch reference values are available, the preference weights derived from the UK population will be used (Gerhards et al., 2011).

<u>Capability Set for Work Questionnaire (CSWQ)</u>
The CSWQ is a valid instrument to measure work capabilities (Abma et al., 2016). The questionnaire explores whether a set of seven valued work aspects, are considered valuable by the worker (A), are enabled in the work context (B), and can be achieved (C). In relation to each of these seven valued work aspects the worker is asked (A) ‘How important is <the value> for you?’ (B) ‘Does your work offer the opportunities to achieve <the value>’ and (C) ‘To what extent do you actually achieve <the value>?’ on a scale from 0 = ‘definitely not’ to 5 = ‘very much’. An individual value is considered part of the capability set of an individual worker when it is considered important (A) (score 4–5), and the workplace offers the opportunity to achieve the value (B) (score 4–5), and the worker is able to achieve the value (C) (score 4–5). The capability set can therefore encompass up to seven values. The combined score of all items results in a total score for the capability set. An overall question for the capability for work was posed: ‘Taking all things together, I think I have enough opportunities to remain working’, which required a response ranging from 1 = ‘totally disagree’ to 5 = ‘ totally agree’ (van Gorp et al., 2018).
The CSWQ will only be administered in patients that are employed (including self-employed patients). It is not applicable for persons that are not employed.

6.1.3 Other study parameters

A number of qualitative measures will be used for the following research objectives:

- To develop rehabilitation care for patients with NMD that is based on the capability approach (a more detailed description can be found in chapter 5.1.2)
- To assess treatment fidelity in designing capability care, training of the health care professionals in providing capability care, delivery of capability care, and receipt and enactment of capability care by the patients with NMD.
- To determine how capability care, in comparison to usual care, facilitates changes in resources and conversion factors, enlarging functionings and capabilities.

Relating this to the MRC framework for process evaluation (Moore et al., 2014, 2015), the first objective relates to the development of the intervention, the second objective refers to the implementation process, whereas the third objective relates to both the mechanisms of impact and to the context.

We will use a selection of commonly used quantitative and qualitative methods listed by the MRC for process evaluation (Moore et al., 2014), such as non-participant observations, analysis of routine data, self-report questionnaires, one-to-one interviews and focus groups. This data will be collected through:

- Consultations with the health care professionals
- Multidisciplinary team meetings
- Medical reports
- Questionnaire on resources and personal characteristics
- Interview on experiences with provided care (with patients and if possible their partners)
- Focus groups with health care professionals

<u>Consultations with the health care professionals</u>
Audio recordings of the consultations (in usual care and capability care) with the health care professionals will be made. These recordings will be used to evaluate the delivery of capability care (an aspect of treatment fidelity) and in what respect capability care differs from usual care. They will be analysed in terms of the capability approach and based on the seven capability dimensions used by Alkire (2002). These dimensions are: (1) Life, health; (2) Knowledge; (3) Aesthetic experience; (4) Play; (5) Sociability (friendship); (6) Practical reasonableness; (7) Religion or transcendence. The analysis is described in more detail in paragraph 8.3.

<u>Multidisciplinary team meetings</u>
Audio recordings of the multidisciplinary team meetings (in usual care and capability care) will be made. In these team meetings, the findings of all health care professionals are discussed, which leads to a decision on how to continue the rehabilitation for each patient. These recordings will be used to evaluate the delivery of capability care (an aspect of treatment fidelity) and in what respect capability care differs from usual care. They will be analysed in terms of the capability approach and based on the seven capability dimensions used by Alkire (2002). These dimensions are: (1) Life, health; (2) Knowledge; (3) Aesthetic experience; (4) Play; (5) Sociability (friendship); (6) Practical reasonableness; (7) Religion or transcendence. The analysis is described in more detail in paragraph 8.3.

<u>Medical reports</u>
All medical reports (including reports from e.g. allied health professionals and nurses) from the departments of neurology and rehabilitation during the inclusion period of the patient will be analysed, to determine the delivery of capability care (an aspect of treatment fidelity) and in what respect capability care differs from usual care. The medical reports will be obtained from the electronic patient file (EPIC) and will be analysed in terms of the capability approach and based on the seven capability dimensions used by Alkire (2002). These dimensions are: (1) Life, health; (2) Knowledge; (3) Aesthetic experience; (4) Play; (5) Sociability (friendship); (6) Practical reasonableness; (7) Religion or transcendence. The analysis is described in more detail in paragraph 8.3.

<u>Questionnaire on resources and personal characteristics (self-developed)</u>
A short questionnaire has been developed (see appendix F) to determine resources at patients' disposal (e.g. income, formal and informal care and the use of aids). This

information will be analysed quantitatively (to describe the patient group) and qualitatively (to triangulate with the results from the interviews). In capability terminology, the information from these questionnaires will provide insight into resources.

Interview on experiences with provided care (at follow-up)
At follow-up, a retrospective interview with the patients and if possible with their partners will be conducted.
This interview focuses on how the patients (and their partners) have experienced the health care and how this has changed several aspects in their lives, and on the limitations and barriers that prevent making these changes (see interview guide in appendix F; outcome measures). If partners are willing to participate in the interview, a separate interview with the partner will be conducted (see interview guide in appendix F). Interviews will be audio-recorded.

Interviews will be held and analysed in parallel until data saturation has been reached. This point is reached when the experiences do not provide new insights. When collecting and analysing in parallel, this ensures that emerging themes from earlier interviews can be investigated in more depth in later interviews (Moore et al., 2014). It is expected that per group (usual and capability care groups), between 10 and 20 patients and if possible their partners will be interviewed.

Focus groups with health care professionals
After the capability care group has completed their rehabilitation treatment, focus groups will be organised with the health care professionals to discuss their experiences with delivering capability care. Depending on the exact number of health care professionals involved in the study, we will organise around 5 focus groups with 4-6 health care professionals per group. The topics introduced during the focus groups by the research team are: the capability care training, the delivery of capability care during rehabilitation treatment of different patients, the receipt of capability care by the patients, the contextual factors involved that enable or prohibit the delivery of capability care, and the differences with usual care. Focus group sessions will be audio-recorded.

6.2 Randomisation, blinding and treatment allocation

The study design is a controlled before-after design using a mixed methods approach. We include two patient groups; the first group will receive usual care, the second group will receive capability care. Randomization and blinding is not feasible in the present study, as all the members of the multidisciplinary outpatient rehabilitation care team need to be trained in providing capability care. Therefore, we are using a before-after design with the two groups receiving care at a different point in time. This design is also needed because the development of the capability care intervention will be based upon the experiences and information gathered in providing usual care (as described in chapter 5.1.2). Although we do not explicitly randomize the allocation of patients to treatment groups, we expect it to be close to randomization because the only explicit difference between the groups is the time of inclusion and treatment. Additionally, we will blind the patient to which treatment they are receiving and ask the patient retrospectively whether this blinding has been broken. Given the nature of the medical conditions (FSHD, DM1) and provided care we do not expect any seasonal influences.

The timeline of the study design can be found in the table below.

Table 1. Timeline of the study.

	Year 1		Year 2			Year 3	
Inclusion of patients receiving optimal usual care; baseline measurements	6 m						
Follow up of patients in usual care group (using mixed methods)		6 m					
Capability training of multidisciplinary healthcare team			3 m				
Inclusion of patients receiving capability care; baseline measurements				6 m			
Follow up of patients receiving capability care (using mixed methods)					6	m	

6.3 Study procedures

All subjects will undergo the measurements as listed in the tables below (table 2 and 3) during the baseline measurement (T0) and 6 months after the baseline measurement (T1). Subjects in both groups (usual care and capability care) will undergo the same assessment procedure. For all questionnaires, the preferred method of assessment is online. The cloud-based clinical data platform CastorEDC will be used for this. Participants will receive an invitation with an online link in their email. If a participant prefers administration with pen and paper, this is also possible. In that case, questionnaires will be sent by post.

There are two exceptions when participants will not undergo all assessments:

- The CSWQ will only be administered when a participant is employed, including self-employment.
- The interview on the patient's experience with the provided health care is expected to be conducted with 10-20 participants per group and if possible their partners.

The COPM is a semi-structured interview that will be conducted by research assistants. These assistants are experienced occupational therapists that are familiar with using the COPM. The interview will be conducted by phone or a video call.

The interviews on experiences with provided care will be conducted by researchers or research assistants. An interview guide (appendix F) will be used. Furthermore, researchers and research assistants will discuss their experiences with conducting the interviews. Analysis of the audio recordings of interviews will be conducted by an independent researcher (someone that has not conducted the particular interview).

Table 2. Overview of baseline measurements.

T0: Outcome measure	Method of assessment	Duration	Assessor
COPM	Semi-structured interview	60 minutes	Research assistant
ICECAP-A	Questionnaire	5 minutes	Online by patient
USER-P	Questionnaire	10 minutes	Online by patient
EQ-5D-5L	Questionnaire	5-10 minutes	Online by patient
SF-36	Questionnaire	10 minutes	Online by patient
CSWQ	Questionnaire	10 minutes	Online by patient
Questionnaire on resources and personal characteristics	Questionnaire	5-10 minutes	Online by patient

Table 3. Overview of follow-up measurements.

T1: Outcome measure	Method of assessment	Duration	Assessor
COPM – re-scoring	Semi-structured interview	5 minutes	Research assistant
Interview on experiences with provided care	Semi-structured interview	55 minutes (and if possible 55 minutes with partner)	Researcher (BB, EP) or research assistant
ICECAP-A	Questionnaire	5 minutes	Online by patient
USER-P	Questionnaire	10 minutes	Online by patient
EQ-5D-5L	Questionnaire	5-10 minutes	Online by patient
SF-36	Questionnaire	10 minutes	Online by patient
CSWQ	Questionnaire	10 minutes	Online by patient
Questionnaire on resources and personal characteristics	Questionnaire	5-10 minutes	Online by patient

6.4 Withdrawal of individual subjects

Subjects can leave the study at any time for any reason if they wish to do so without any consequences. The investigator can decide to withdraw a subject from the study for urgent medical reasons.

6.4.1 Specific criteria for withdrawal

Not applicable.

6.5 Replacement of individual subjects after withdrawal

When a subject withdraws from the study, it will not be replaced. With an inclusion of 30 subjects per group, if a maximum of 6 participants per group withdraw, the expected effect of

the intervention will still show a significant effect (see power calculation as described in chapter 4.4).

6.6 Follow-up of subjects withdrawn from treatment

Subjects that withdraw from the study will be forwarded for aftercare to their general practitioner, neurologist or rehabilitation physician if needed. Depending on the participant's need, referral to the right health care provider will be arranged. Subjects can also withdraw from the study without terminating their multidisciplinary treatment. In that case, the health care providers will be notified of the subject's withdrawal.
The data collected from subjects that have withdrawn from the study will not be used, as this will only consist of baseline measurements when a subject has withdrawn. With the present study design, consisting of only two measurement moments, it is not possible to use an intention-to-treat analysis for the subjects that have withdrawn. However, the number of withdrawals and reason for withdrawal (if the patient is prepared to share his reason) will be described. With the collected baseline-data and reasons for withdrawal, we will analyse whether there is a certain pattern to be found for patients withdrawing from the study.

6.7 Premature termination of the study

In case of any unforeseen, prematurely termination of the study, all participants will be informed by the researcher. Participants can indicate if they agree on storage of the data obtained until that point.

7. SAFETY REPORTING

7.1 Temporary halt for reasons of subject safety

In accordance to section 10, subsection 4, of the WMO, the sponsor will suspend the study if there is sufficient ground that continuation of the study will jeopardise subject health or safety. The sponsor will notify the accredited METC without undue delay of a temporary halt including the reason for such an action. The study will be suspended pending a further positive decision by the accredited METC. The investigator will take care that all subjects are kept informed.

7.2 AEs, SAEs and SUSARs

7.2.1 Adverse events (AEs)

Adverse events are defined as any undesirable experience occurring to a subject during the study, whether or not considered related to the experimental intervention. All adverse events reported spontaneously by the subject or observed by the investigator or his staff will be recorded.

7.2.2 Serious adverse events (SAEs)

A serious adverse event is any untoward medical occurrence or effect that

- results in death;
- is life threatening (at the time of the event);
- requires hospitalisation or prolongation of existing inpatients' hospitalisation;
- results in persistent or significant disability or incapacity;
- is a congenital anomaly or birth defect; or
- any other important medical event that did not result in any of the outcomes listed above due to medical or surgical intervention but could have been based upon appropriate judgement by the investigator.

An elective hospital admission will not be considered as a serious adverse event.

The investigator will report all SAEs to the sponsor without undue delay after obtaining knowledge of the events, except for SAEs that are not considered related to the experimental intervention.

The sponsor will report the SAEs through the web portal *ToetsingOnline* to the accredited METC that approved the protocol:

- within 7 days of first knowledge for SAEs that result in death or are life threatening followed by a period of maximum of 8 days to complete the initial preliminary report.
- within a period of maximum 15 days after the sponsor has first knowledge of the serious adverse events for all SAEs considered related to the experimental intervention.
- in the annual progress report for all SAEs not considered related to the experimental intervention.

7.2.1 Suspected unexpected serious adverse reactions (SUSARs)

Not applicable.

7.3 Follow-up of adverse events

All AEs will be followed until they have abated, or until a stable situation has been reached. Depending on the event, follow up may require additional tests or medical procedures as indicated, and/or referral to the general physician or a medical specialist.
SAEs need to be reported till end of study within the Netherlands, as defined in the protocol.

8. STATISTICAL ANALYSIS

Data and derived parameters

The data collected in this study will be both qualitative and quantitative data. An overview is presented in the table below (table 4), showing all the outcome measures, their level of measurement and derived parameters. In the paragraphs below, the statistical analysis of the outcome measures, and how they contribute to answering the study objectives, is described in more detail. An overview of all the (statistical) analyses can be found in table 5.

Table 4. Primary and secondary outcome measures, their characteristics, and derived parameters.

Primary outcome measure	COPM	Quantitative	1. Mean scores of 1-10 range for COPM-P (interval level of measurement).<br>2. Mean scores of 1-10 range for COPM-S (interval level of measurement).
Secondary outcome measures	ICECAP-A	Quantitative	1. Descriptive system (5 domains, 4 levels) (ordinal level of measurement).<br>2. Non-weighted sum scores, calculated by summing ICECAP-A item scores (interval level of measurement).<br>3. Total index score obtained by summing preference-based weights (tariff scores) assigned to all different levels, ranging from 0 (no capabilities) to 1 (full capability) (ratio level of measurement).
	USER-P	Quantitative	1. Frequency scale; likert scale score 0-5, sum score converted to 0-100 (interval level of measurement).<br>2. Restriction scale; likert scale score 0-3, sum score converted to 0-100 (interval level of measurement).<br>3. Satisfaction scale; likert scale score 0-4, sum score converted to 0-100 (interval level of measurement).
	SF-36	Quantitative	1. Scale scores for the 8 domains, likert scales converted to 0-100 (interval level of measurement)<br>o physical functioning (PF),<br>o role limitations due to physical health problems (RP),<br>o bodily pain (BP),<br>o general health perceptions (GH),<br>o vitality (VT),<br>o social functioning (SF),<br>o role limitations due to emotional problems (RE),<br>o general mental health (MH).<br>2. SF-6D scores derived from SF-

			36 scores (ratio level of measurement).<br>3. SF-6D preference-based total index score (ratio level of measurement).
	EQ-5D-5L	Quantitative	1. Descriptive system (5 domains, 5 levels) (ordinal level of measurement).<br>2. Total index score, ranging from 0 (death) to 1 (full health) (ratio level of measurement).
	CSWQ	Quantitative	1. Total score for the capability set (range 0-7; based on the questions calculated whether a capability is present yes/no) (interval level of measurement)<br>2. Overall question likert scale range 1-5 (ordinal level of measurement)
Other	Consultations with health care professionals (audio recorded)	Qualitative and quantitative	Qualitative information<br>Capability scoring form
	Multidisciplinary team meetings (audio recorded)	Qualitative and quantitative	Qualitative information<br>Capability scoring form
	Medical reports	Qualitative and quantitative	Qualitative information<br>Capability scoring form
	Questionnaire on resources and personal characteristics	Quantitative and qualitative	Descriptive data (nominal and ordinal)
	Interview on experiences with provided care (audio recorded)	Qualitative	Qualitative information
	Focus groups with health care professionals (audio recorded)	Qualitative	Qualitative information

Table 5. Overview of the (statistical) analyses per research objective.

Objective	Outcome measures	Analysis	Statistical test
**To compare the quality of life of patients with NMD receiving capability care versus usual care**	COPM usual care<br>COPM capability care	COPM-P usual care T1 vs. COPM-P capability care T1 (T0 scores as covariate)<br>COPM-S usual care T1 vs. COPM-S capability care T1 (T0 scores as covariate)	ANCOVA

**To develop rehabilitation care for patients with NMD that is based on the capability approach**	Consultations with health care professionals<br>Multidisciplinary team meetings<br>Medical reports<br>Interview on experiences with provided care	Qualitative analysis	N/A
**To assess treatment fidelity in designing capability care, training of the health care professionals in providing capability care, delivery of capability care, and receipt and enactment of capability care by the patients with NMD**	Consultations with health care professionals<br>Multidisciplinary team meetings<br>Medical reports<br>Interview on experiences with provided care<br>Focus groups with health care professionals	Qualitative analysis<br>Capability scoring form (to be developed)	N/A<br>To be determined
**To determine how capability care, in comparison to usual care, facilitates changes in resources and conversion factors, enlarging functionings and capabilities**	Consultations with health care professionals<br>Medical reports<br>Questionnaire on resources and personal characteristics<br>Interview on experiences with provided care<br>Focus groups with health care professionals	Qualitative analysis	N/A
**To compare the capabilities outcome of patients with NMD receiving capability care versus usual care, using the ICECAP-A that**	ICECAP-A usual care<br>ICECAP-A capability care	Non-weighted ICECAP-A sum scores usual care T1 vs capability care T1 (T0 scores as covariate)	ANCOVA

**covers five attributes (e.g. attachment, stability, achievement, enjoyment, autonomy) of wellbeing**			
**To compare the participation outcome (USER-P) of patients with NMD receiving capability care versus usual care**	USER-P usual care<br>USER-P capability care	USER-P usual care T1 vs. USER-P capability care T1 (T0 scores as covariate)	ANCOVA
**To compare the work capabilities outcome (CSWQ) of patients with NMD receiving capability care versus usual care**	CSWQ usual care<br>CSWQ capability care	CSWQ total score usual care T1 vs. CSWQ total score capability care T1 (T0 scores as covariate)<br><br>CSWQ overall question (T1-T0) usual care vs capability care	ANCOVA<br><br>Mann Whitney U test
**To determine whether the use of a broader measure of quality of life (ICECAP-A), compared to a health-related quality of life measure (EQ-5D-5L, SF-36), leads to other outcomes in terms of assessing the benefits of care**	ICECAP-A usual care<br>ICECAP-A capability care<br><br>EQ-5D-5L usual care<br>EQ-5D-5L capability care<br><br>SF-36 usual care<br>SF-36 capability care	Multiple comparisons between usual care group and capability care group on difference T1-T0.	ANCOVA, contingency table

Handling of missing data

The data collected from subjects that have withdrawn from the study will not be used, as this will only consist of baseline measurements when a subject has withdrawn. With the present study design, consisting of only two measurement moments, it is not possible to use an intention-to-treat analysis for the subjects that have withdrawn. However, the number of withdrawals and reason for withdrawal (if the patient is prepared to share his reason) will be described. With the collected baseline-data and reasons for withdrawal, we will analyse whether there is a certain pattern to be found for patients withdrawing from the study.
Missing data from incomplete measures will be excluded from the analyses. However, this subject will not be completely excluded from all analyses. For example, when the subject has completed all measurement moments for the primary outcome measure but not for one of the secondary outcome measures, we will include the data for the primary outcome measure in the analysis, but exclude the data for the incomplete secondary outcome measure. Or, if the primary outcome measure is missing but the secondary outcome measures are complete, we

will exclude the data for the primary outcome measure, but include the data for the secondary outcome measures.

8.1 Primary study parameters

Primary outcome measure

The primary outcome measure is the COPM. The COPM performance (COPM-P) and satisfaction (COPM-S) scales provide continuous data.

For the COPM, the performance score (COPM-P) and satisfaction score (COPM-S) are derived from the scores on the individual problems in activities, divided by the number of activities:

$$COPM\ performance\ score\ (COPM\text{-}P) = \frac{sum\ score\ performance}{number\ of\ activities}$$

$$COPM\ satisfaction\ score\ (COPM\text{-}S) = \frac{sum\ score\ satisfaction}{number\ of\ activities}$$

Statistical analysis

Data will be visualised using scatter-plots, box plots and histograms. Descriptive statistics will include mean and standard deviation. To answer the primary research question, whether there is a difference in improvement at T1 between the capability care and usual care group, an ANCOVA model will be fitted. Covariates will be the T0 scores of both groups, age, gender and diagnosis. With the model it is tested whether an intervention-effect is present. For an intervention-effect to be present, the scores on both the COPM-P and COPM-S will need to show a significant change. A p-value below 0.05 will be considered significant.

8.2 Secondary study parameters

For the following secondary study parameters, similar analyses as described above (ANCOVA model) will be performed:

- ICECAP-A preference-weighted index score
- ICECAP-A non-weighted sum score
- USER-P
- CSWQ total score
- EQ-5D-5L preference-weighted index score
- EQ-5D-5L non-weighted sum score
- SF-36 preference-weighted index score
- SF-36 non-weighted sum score

The CSWQ overall question will be analysed using a Mann-Whitney U test. Median and range will be described. The Mann Whitney U test will be performed on the difference score between T1 and T0, to determine whether there is a difference between the usual care and capability care group. A p-value below 0.05 will be considered significant.

Comparison of ICECAP-A, EQ-5D-5L and SF-36 outcomes

As described above, ANCOVA models will be fitted to determine whether patients significantly improved (a p-value below 0.05) in terms of ICECAP-A, EQ-5D-5L and SF-36 scores. Multiple contingency tables will be created to compare the number of patients that

are responder or non-responder based on the ICECAP-A, EQ-5D-5L and SF-36 ANCOVA models. These comparisons will be performed both for the non-weighted scores and the preference-weighted scores. An analysis solely based on the preference-weighted scores may conclude that the measure is not responsive, when in fact, it could be that the descriptive system of the measure is responsive but the change is not valued.

To calculate the EQ-5D-5L preference-weighted scores the Dutch reference values will be used (Versteegh et al., 2016).

ICECAP-A preference-weighted scores will be calculated using the algorithm from Flynn et al. (Flynn et al., 2015).

Based on a selection of SF-36 items, the SF-6D can be derived. The SF-6D is composed of six multi-level dimensions, and any patient who completes the SF-36 can be uniquely classified according to the SF-6D. The SF-6D provides a means for using the SF-36 in health economic evaluation by estimating a preference-based index value based on preference weights obtained from a sample of the general population. Because no Dutch reference values are available, the preference weights derived from the UK population will be used (Gerhards et al., 2011).

In addition, an anchor-based analysis will be performed to explore whether ICECAP-A scores change in the expected direction as indicated by changes in the EQ-5D-5L and SF-36 scores. Both the EQ-5D-5L and SF-36 scores will be used to divide the patients into groups: (1) patients that worsened; (2) patients that showed no significant change; (3) patients that improved; and within these groups the mean ICECAP-A changes will be used to see whether they show the same direction of change (Keeley et al., 2015).

8.3 Other study parameters

The other study data collected will be used for the development of capability care and for the process evaluation. Figure 4 (see chapter 6) gives an overview of the process evaluation. In this chapter the analyses that will be performed are described.

8.3.1 Developing capability care

For the development of capability care (as described in chapter 5.1.2), a qualitative analysis will be performed on data collected in the usual care group. The data that will be used are the consultations with the health care professionals (audio recorded), the multidisciplinary team meetings (audio recorded), the medical reports (from the patient files) and the interview with patients (and partners) at 6 months follow up.
The consultations with health care professionals and the multidisciplinary team meetings will be audio recorded. A random selection of these recordings will be transcribed; relevant fragments will be selected by two researchers independently and discussed until agreement has been reached. If the researchers find a large variation between the selected records, a new random selection will be added for analysis to determine whether this is a true variation or a variation because of the selection process. The medical reports are written; they will be obtained from the electronic patient record system (EPIC) that is used at Radboudumc. Relevant information from the medical reports will be selected by two researchers independently and discussed until agreement has been reached.

The qualitative analysis of the selected fragments and medical reports consists of the following steps:

1) Codes will be allocated to meaningful text parts answering the research questions by two independent researchers. This coding will be done using the program Atlas-Ti.
2) The researchers will discuss codes and categories.
3) The codes and categories are discussed with the whole research group and overarching themes may be discussed.
4) Themes and subthemes are formulated and illustrated with original quotes.

For the interviews with patients and, if possible, their partners, a constant comparative method (Evers, 2016) will be applied using the following alternating steps:

1) 3 to 5 interviews will take place with patients and partners and will be audio-recorded
2) The interviews will be transcribed ad verbatim and field notes of the relevant observations from the audio recordings will be made. After reading the interviews, codes will be allocated to meaningful text parts answering the research questions. This coding will be done using the program Atlas-Ti.
3) The researchers will discuss codes and insights and discuss whether new insights are topics to be addressed in the next interviews.
4) Another 3-5 interviews are being held and transcribed, coded and discussed and some initial categories are being discussed.
5) The final 5-10 interviews are being held, transcribed and coded and evaluated whether these confirm the preliminary categories or whether new categories arise. New patients and partners are interviewed and coded and categorized until no new categories arise.
6) The codes and categories are discussed with the whole research group and overarching themes may be discussed.
7) Final themes and subthemes are formulated and illustrated with original quotes.

The information obtained through qualitative analysis will be used for discussions within the project team, with health care providers and with experts from the capability approach field to develop capability care (as described in chapter 5.1.2).

8.3.2 Assessment of treatment fidelity

An intervention may have limited effects or positive outcomes because of weaknesses in its design or because it is not properly implemented. Therefore, a process evaluation should capture fidelity: was the intervention delivered as intended?

To assess treatment fidelity (delivery of capability care and receipt and enactment of capability care), both a qualitative and quantitative analysis will be performed. Information on delivery of capability care will be derived from the audio recordings of consultations andteam meetings, medical reports, and focus groups with healthcare professionals. Information on receipt and enactment of capability care will be derived from the interview with patients and, if possible, partners.

The method for the qualitative part of the analysis to assess treatment fidelity has been described above (chapter 8.3.1.); however, when using this information for assessment of treatment fidelity, we will use data from both the usual care and capability care group to conduct the analysis as described above. The information obtained through this analysis will

provide the information needed to assess treatment fidelity qualitatively because it retrieves and analyses information on the delivery, receipt and enactment of care.

Capability scoring form
The quantitative part of the analysis to assess treatment fidelity (delivery of capability care) will be performed as described below.
When developing capability care, a scoring form for delivery of capability care will be developed. This 'capability scoring form' will include treatment elements based on the capability approach (e.g. resources, conversion factors, functionings) and the seven capability dimensions used by Alkire (2002). These dimensions are: (1) life, health; (2) knowledge; (3) aesthetic experience; (4) play; (5) sociability (friendship); (6) practical reasonableness; (7) religion or transcendence.
The elements in this form will be classified into (1) essential and unique; (2) essential but not unique; (3) compatible but not essential and not unique; (4) prohibited; and (5) unique but not essential (Leeuw, Goossens, de Vet, & Vlaeyen, 2009). A scoring form can be constructed by scoring the treatment elements on whether or not they did occur, or on how often they did occur (e.g. on a likert scale or counting of elements). Depending on the development of capability care, it will be determined which treatment elements will be included and what scoring system is most suitable for these elements.

Two blinded research assistants will independently score a random selection (from usual care and capability care) of the consultations with the health care professionals, multidisciplinary team meetings and medical reports using the 'capability scoring form'. Interrater reliability, protocol adherence, treatment contamination and treatment differentiation (Leeuw et al., 2009) will be calculated. The exact calculation method needs to be determined based on the scoring system used (e.g. dichotomous scoring, likert scale, counting of elements). Besides assessing treatment fidelity, the obtained scores will also be used to determine the extent to which usual care and capability care differ.

8.3.3 Exploring how capability care facilitates changes in resources, conversion factors, functionings and capabilities

Exploring the mechanisms through which an intervention brings about change is crucial to understanding how the effects of the intervention were realized and how these effects may be replicated by future implementations. Given that our intervention is based on the capability approach, which assumes that well-being should be understood in terms of capabilities and that these are determined by a dynamic interaction between access to *resources*, *conversion factors,* and *opportunities,* we aim to explore these potential mechanisms of impact. To determine how capability care, in comparison to usual care, facilitates changes in resources and conversion factors, enlarging functionings and capabilities (and in which dimensions these are enlarged), a qualitative comparison will be done between both groups, as described below.

The audio recordings of consultations with healthcare professionals and the multidisciplinary team meetings, as well as the interviews with patients and partners, will be analysed as described in 8.3.1 with a focus on mechanisms of impact. The information from the questionnaire on resources and personal characteristics will be used next to the information from the medical reports to determine the resources that patients use and whether this has changed during the treatment/follow-up period.

The focus groups with health care professionals will be audio recorded. A qualitative analysis will be performed using the following steps:

1) The focus groups will be transcribed at verbatim and field notes of the relevant observations during the focus groups will be made. After reading the transcriptions, codes will be allocated to meaningful text parts answering the research questions. This coding will be done using the program Atlas-Ti.
2) The researchers will discuss codes and categories.
3) The codes and categories are discussed with the whole research group and overarching themes may be discussed.
4) Themes and subthemes are formulated and illustrated with original quotes.

The credibility of the findings is supported by triangulation of the findings from the interviews (patients and partners), focus groups with health care professionals, consultations, multidisciplinary team meetings, medical reports and questionnaires on resources and personal characteristics.

8.3.4 Exploring how the context influences the implementation, mechanisms and outcomes of capability care

To explore the influence of the context on the implementation, mechanisms of impact, and outcomes of capability care, we will use qualitative information obtained from the interviews with the patients (and partners) and from the focus groups with the health care professionals. This information will be analysed qualitatively as described in chapter 8.3.1 and 8.3.3 with a focus on context.

8.4 Interim analysis

Not applicable.

9. ETHICAL CONSIDERATIONS

9.1 Regulation statement

This study will be conducted according to the principles of the Declaration of Helsinki, version 2013 and in accordance with the Medical Research Involving Human Subjects Act (WMO). The study will only proceed after approval of the Ethical Committee. Ethical approval for the study will be requested at the Ethical Committee of Arnhem/Nijmegen.

9.2 Recruitment and consent

Recruitment and consent will be obtained following the next procedure:

1. Eligible patients will be pre-screened based on diagnosis, comorbidities and age by their neurologist and/or rehabilitation physician. Eligible patients will be informed by their neurologist and/or rehabilitation physician about the study and will receive the information flyer. Patients will be asked whether their contact details can be forwarded to the researchers, so that the researchers can contact them over the phone with more information about the study.
2. The researcher (BB or EP) contacts the patient over the phone to explain more about the study, and will send the patient information form if the patient is still interested in participating after this phone call.
3. After at least one week, the researcher contacts the patient over the phone again to ask whether he has additional questions and wishes to participate in the study. If so, the patient is asked to sign the informed consent and return this via post.
4. Once the informed consent has been received, the researcher will sign the form and return a copy via post to the participant.
5. An appointment with the research assistant is planned for the first measurement (prior to start of the rehabilitation treatment). Before starting the measurement, the research assistant will check whether the patient meets all inclusion criteria. We do not expect a large number of exclusions, as the patient has been pre-screened by their physician.

9.3 Benefits and risks assessment, group relatedness

The participants in the intervention group receive healthcare based on the capability approach (capability care). It is expected that this type of healthcare is more person-centred and will lead to a higher quality of life for patients with neuromuscular disease. The provision of rehabilitation care with an explicit focus on strengthening patients' capabilities is not expected to incur greater risk than treatment as usual.

Participants in both the usual care and capability care groups undergo two measurement moments. Measurement moments contain an interview (1 hour) and self-report questionnaires (electronically or on paper) (50 minutes). The interview will take place at the Radboudumc or at a location convenient for the participant (at home) so that travel time is limited. The measurements are non-invasive and no health risks are expected. Participants possibly are confronted with their limitations by the questionnaires or during the interviews. The research assistants are trained occupational therapists who can address concerns of participants or advise participants to visit other health professionals.

9.4 Compensation for injury

The sponsor/investigator has a liability insurance which is in accordance with article 7 of the WMO.

The sponsor (also) has an insurance which is in accordance with the legal requirements in the Netherlands (Article 7 WMO). This insurance provides cover for damage to research subjects through injury or death caused by the study.
The insurance applies to the damage that becomes apparent during the study or within 4 years after the end of the study.

9.5 Incentives

Participants will not receive any payment for participation in this study. Participants will be reimbursed for the additional travel costs they make for participating in this study. This will only include travel for the measurements, not for the appointments with their health professionals (as this is usual care).

10. ADMINISTRATIVE ASPECTS, MONITORING AND PUBLICATION

10.1 Handling and storage of data and documents

Data will be coded by an identification code replacing any personal identification data. Coded data will be stored in computer files in a password protected file or in a locked cabinet. The passwords or keys will be kept in a separate locked cabinet or computer file. Only the researchers (BB and EP), project leader (EC) and the research assistants have the authority to decode the data if needed. The key to the codes will be kept in a separate locked computer file. The codes will not contain any information that can lead to any identifiable information.

The audio recordings will be transcribed. The transcribed data will be coded by a random identification code and will be handled with the same procedure as described above. The original audio recordings will be stored in password protected computer files and deleted from the recording device.
The medical reports from the departments of rehabilitation and neurology during the study period will be extracted from the electronic patient file (EPIC) and saved with the random identification code (all personal identification data will be removed). This data will be handled with the same procedure as described above.

After all data is entered into the database and is validated, the database will be locked. Data will be kept according to research guidelines for a period of fifteen years in a secured data management system. Only the researcher, research assistants, statistician and the research group can access the data. Below (table 6) are the rights for each member. The data will be secured by password access.

Table 6. Overview of rights related to access of collected data.

	Access	Data entry	Decode data	Re-identify participants	Aggregate data	Analyse data
Research assistants	x	x	x			
Researchers	x	x	x		x	x
Project leader	x		x		x	x
Research team	x				x	x
Statistician	x				x	x

10.2 Monitoring and Quality Assurance

Study monitoring will take place. A qualified monitor from the Radboudumc Technology Centre Clinical Studies will monitor the study. Monitoring will take place in accordance with the guidelines of the NFU (as described in the document “Kwaliteitsborging mensgebonden onderzoek 2019”). A monitoring plan and data management plan will be written before

including the first participant, in accordance with Radboudumc regulations to obtain permission to start the study (local feasibility application).
The risk of the study is classified as negligible risk.

10.3 Amendments

Amendments are changes made to the research after a favourable opinion by the accredited METC has been given. All amendments will be notified to the METC that gave a favourable opinion.

All substantial amendments will be notified to the METC and to the competent authority.

Non-substantial amendments will not be notified to the accredited METC and the competent authority, but will be recorded and filed by the sponsor.

10.4 Annual progress report

The sponsor/investigator will submit a summary of the progress of the trial to the accredited METC once a year. Information will be provided on the date of inclusion of the first subject, numbers of subjects included and numbers of subjects that have completed the trial, serious adverse events/ serious adverse reactions, other problems, and amendments.

10.5 Temporary halt and (prematurely) end of study report

The investigator/sponsor will notify the accredited METC of the end of the study within a period of 8 weeks. The end of the study is defined as the last patient's last visit.

The sponsor will notify the METC immediately of a temporary halt of the study, including the reason of such an action.

In case the study is ended prematurely, the sponsor will notify the accredited METC within 15 days, including the reasons for the premature termination.

Within one year after the end of the study, the investigator/sponsor will submit a final study report with the results of the study, including any publications/abstracts of the study, to the accredited METC.

10.6 Public disclosure and publication policy

The study will be registered at trialregister.nl before recruiting the first participant.
A design article of the intervention study will be published before analysis of the data. Results of the study will be published in relevant peer-reviewed journals. Lay versions of the publications will be sent to the participants and presented to the Dutch association for Neuromuscular Diseases (Spierziekten Nederland) and the subsidising partner Prinses Beatrix Spierfonds.
The final report will contain a scientific summary and a lay summary with the results of the studies. This final report will be submitted through toetsingonline.nl. Through the CCMO register the results will be publicly available.

11. REFERENCES

Aaronson, N. K., Muller, M., Cohen, P. D., Essink-Bot, M. L., Fekkes, M., Sanderman, R., . . . Verrips, E. (1998). Translation, validation, and norming of the Dutch language version of the SF-36 Health Survey in community and chronic disease populations. *J Clin Epidemiol, 51*(11), 1055-1068. doi:10.1016/s0895-4356(98)00097-3

Abma, F. I., Brouwer, S., de Vries, H. J., Arends, I., Robroek, S. J., Cuijpers, M. P., . . . van der Klink, J. J. (2016). The capability set for work: development and validation of a new questionnaire. *Scand J Work Environ Health, 42*(1), 34-42. doi:10.5271/sjweh.3532

Al-Janabi, H., Flynn, T. N., & Coast, J. (2012). Development of a self-report measure of capability wellbeing for adults: the ICECAP-A. *Qual Life Res, 21*(1), 167-176. doi:10.1007/s11136-011-9927-2

Alkire, S. (2002). *Valuing Freedoms.* Oxford: Oxford University Press.

Borm, G. F., Fransen, J., & Lemmens, W. A. (2007). A simple sample size formula for analysis of covariance in randomized clinical trials. *J Clin Epidemiol, 60*(12), 1234-1238. doi:10.1016/j.jclinepi.2007.02.006

Bos, I., Kuks, J. B., & Wynia, K. (2015). Development and testing psychometric properties of an ICF-based health measure: The Neuromuscular Disease Impact Profile. *J Rehabil Med, 47*(5), 445-453. doi:10.2340/16501977-1938

Deenen, J. C., Arnts, H., van der Maarel, S. M., Padberg, G. W., Verschuuren, J. J., Bakker, E., . . . van Engelen, B. G. (2014). Population-based incidence and prevalence of facioscapulohumeral dystrophy. *Neurology, 83*(12), 1056-1059. doi:10.1212/wnl.0000000000000797

EuroQol Research Foundation. (2019). *EQ-5D-5L User Guide*. Retrieved from https://euroqol.org/publications/user-guides

Evers, J. (2016). *Kwalitatieve analyse: kunst én kunde*. Amsterdam: Boom uitgevers Amsterdam.

Eyssen, I. C., Steultjens, M. P., Oud, T. A., Bolt, E. M., Maasdam, A., & Dekker, J. (2011). Responsiveness of the Canadian occupational performance measure. *J Rehabil Res Dev, 48*(5), 517-528. doi:10.1682/jrrd.2010.06.0110

Flynn, T. N., Huynh, E., Peters, T. J., Al-Janabi, H., Clemens, S., Moody, A., & Coast, J. (2015). Scoring the Icecap-a capability instrument. Estimation of a UK general population tariff. *Health Econ, 24*(3), 258-269. doi:10.1002/hec.3014

Gerhards, S. A., Huibers, M. J., Theunissen, K. A., de Graaf, L. E., Widdershoven, G. A., & Evers, S. M. (2011). The responsiveness of quality of life utilities to change in depression: a comparison of instruments (SF-6D, EQ-5D, and DFD). *Value Health, 14*(5), 732-739. doi:10.1016/j.jval.2010.12.004

Harper, P. S. (2001). *Myotonic dystrophy* (3rd ed.). Philadelphia: WB Saunders.

Herdman, M., Gudex, C., Lloyd, A., Janssen, M., Kind, P., Parkin, D., . . . Badia, X. (2011). Development and preliminary testing of the new five-level version of EQ-5D (EQ-5D-5L). *Qual Life Res, 20*(10), 1727-1736. doi:10.1007/s11136-011-9903-x

Landfeldt, E., Edström, J., Jimenez-Moreno, C., van Engelen, B. G. M., Kirschner, J., & Lochmüller, H. (2019). Health-Related Quality of Life in Patients with Adult-Onset Myotonic Dystrophy Type 1: A Systematic Review. *Patient, 12*(4), 365-373. doi:10.1007/s40271-019-00357-y

Law, M., Baptiste, S., Carswell, A., McColl, M. A., Polatajko, H., & Pollock, N. (1998). *Canadian Occupational Performance Measure.* Ottawa: CAOT Publications ACE.

Leeuw, M., Goossens, M. E., de Vet, H. C., & Vlaeyen, J. W. (2009). The fidelity of treatment delivery can be assessed in treatment outcome studies: a successful illustration from behavioral medicine. *J Clin Epidemiol, 62*(1), 81-90. doi:10.1016/j.jclinepi.2008.03.008

Litwin, M. S. (2002). RAND 36-Item Health Survey v2 (SF-36 v2) and UCLA Prostate Cancer Index health-related quality of life scoring instructions. In.

Mitchell, P. M., Roberts, T. E., Barton, P. M., & Coast, J. (2017). Applications of the Capability Approach in the Health Field: A Literature Review. *Soc Indic Res, 133*(1), 345-371. doi:10.1007/s11205-016-1356-8

Mitra, S. (2017). *Disability, health and human development*: Palgrave studies in disability and international development.

Moore, G., Audrey, S., Barker, M., Bond, L., Bonell, C., Hardeman, W., . . . L, B. (2014). *Process evaluation of complex interventions. UK Medical Research Council (MRC) guidance*. Retrieved from

Moore, G., Audrey, S., Barker, M., Bond, L., Bonell, C., Hardeman, W., . . . L, B. (2015). Process Evaluation of Complex Interventions: Medical Research Council Guidance. *BMJ: British medical journal, 350*. doi:10.1136/bmj.h1258

Mul, K., Lassche, S., Voermans, N. C., Padberg, G. W., Horlings, C. G., & van Engelen, B. G. (2016). What's in a name? The clinical features of facioscapulohumeral muscular dystrophy. *Pract Neurol, 16*(3), 201-207. doi:10.1136/practneurol-2015-001353

Okkersen, K., Jimenez-Moreno, C., Wenninger, S., Daidj, F., Glennon, J., Cumming, S., . . . van Engelen, B. G. M. (2018). Cognitive behavioural therapy with optional graded exercise therapy in patients with severe fatigue with myotonic dystrophy type 1: a multicentre, single-blind, randomised trial. *Lancet Neurol, 17*(8), 671-680. doi:10.1016/s1474-4422(18)30203-5

Padberg, G. W. (1982). *Facioscapulohumeral disease.* University of Leiden, Leiden.

Post, M. W., van der Zee, C. H., Hennink, J., Schafrat, C. G., Visser-Meily, J. M., & van Berlekom, S. B. (2012). Validity of the utrecht scale for evaluation of rehabilitation-participation. *Disabil Rehabil, 34*(6), 478-485. doi:10.3109/09638288.2011.608148

Rijke, W. J., Vermeulen, A. M., Wendrich, K., Mylanus, E., Langereis, M. C., & van der Wilt, G. J. (2019). Capability of deaf children with a cochlear implant. *Disabil Rehabil*, 1-6. doi:10.1080/09638288.2019.1689580

Robeyns, I. (2005). The Capability Approach: a theoretical survey. *Journal of Human Development, 6*(1), 93-117. doi:10.1080/146498805200034266

Robeyns, I. (2006). The Capability Approach in Practice*. *Journal of Political Philosophy, 14*(3), 351-376. doi:10.1111/j.1467-9760.2006.00263.x

Robeyns, I. (2017). *Wellbeing, Freedom and Social Justice. The capability approach re-examined.*: Open Book Publishers.

Sen, A. (1992). *Inequality reexamined.* . Boston: Harvard University Press.

Sen, A. (1993). Capability and Well-Being. In Nussbaum & Sen (Eds.), *The Quality of Life*. Oxford: Clarendon Press.

van der Zee, C. H., Baars-Elsinga, A., Visser-Meily, J. M., & Post, M. W. (2013). Responsiveness of two participation measures in an outpatient rehabilitation setting. *Scand J Occup Ther, 20*(3), 201-208. doi:10.3109/11038128.2012.754491

van der Zee, C. H., Post, M. W., Brinkhof, M. W., & Wagenaar, R. C. (2014). Comparison of the Utrecht Scale for Evaluation of Rehabilitation-Participation with the ICF Measure of Participation and Activities Screener and the WHO Disability Assessment Schedule II in persons with spinal cord injury. *Arch Phys Med Rehabil, 95*(1), 87-93. doi:10.1016/j.apmr.2013.08.236

van der Zee, C. H., Priesterbach, A. R., van der Dussen, L., Kap, A., Schepers, V. P., Visser-Meily, J. M., & Post, M. W. (2010). Reproducibility of three self-report participation measures: The ICF Measure of Participation and Activities Screener, the Participation Scale, and the Utrecht Scale for Evaluation of Rehabilitation-Participation. *J Rehabil Med, 42*(8), 752-757. doi:10.2340/16501977-0589

van Engelen, B. (2015). Cognitive behaviour therapy plus aerobic exercise training to increase activity in patients with myotonic dystrophy type 1 (DM1) compared to usual care (OPTIMISTIC): study protocol for randomised controlled trial. *Trials, 16*, 224. doi:10.1186/s13063-015-0737-7

van Gorp, D. A. M., van der Klink, J. J. L., Abma, F. I., Jongen, P. J., van Lieshout, I., Arnoldus, E. P. J., . . . van der Hiele, K. (2018). The capability set for work - correlates of sustainable employability in workers with multiple sclerosis. *Health Qual Life Outcomes, 16*(1), 113. doi:10.1186/s12955-018-0942-7

Veenhuizen, Y., Cup, E. H. C., Jonker, M. A., Voet, N. B. M., van Keulen, B. J., Maas, D. M., . . . Geurts, A. C. H. (2019). Self-management program improves participation in patients with neuromuscular disease: A randomized controlled trial. *Neurology, 93*(18), e1720-e1731. doi:10.1212/wnl.0000000000008393

Versteegh, M. M., Vermeulen, K. M., Evers, S. M. A. A., de Wit, G. A., Prenger, R., & Stolk, E. A. (2016). Dutch Tariff for the Five-Level Version of EQ-5D. *Value Health, 19*(4), 343-352. doi:10.1016/j.jval.2016.01.003

Ware, J. E., Snow, K. K., Kosinski, M., & Gandek, B. (1993). *SF-36 Health Survey Manual and Interpretation Guide.* . Boston: The Health Institute.

Winter, Y., Schepelmann, K., Spottke, A. E., Claus, D., Grothe, C., Schröder, R., . . . Dodel, R. (2010). Health-related quality of life in ALS, myasthenia gravis and facioscapulohumeral muscular dystrophy. *J Neurol, 257*(9), 1473-1481. doi:10.1007/s00415-010-5549-9
