# Supplementary material for: Care for capabilities: Implementing the capability approach in rehabilitation of patients with neuromuscular diseases. Study protocol of the controlled before-after ReCap-NMD study
Source: PLoS One. 2021 Dec 21;16(12):e0261475. doi: 10.1371/journal.pone.0261475 (PMC8691629; doi:10.1371/journal.pone.0261475)
Supplement: Peer review history

# Editor report: Decision Letter 0

**DOI:** 10.1371/journal.pone.0261475.r001

**Authors:** Thomas Phillips

3 Dec 2021

Care for capabilities: implementing the capability approach in rehabilitation of patients with neuromuscular diseases. Study protocol of the controlled before-after ReCap-NMD study.

PONE-D-21-32625

Dear Dr. Bloemen,

We’re pleased to inform you that your manuscript has been judged scientifically suitable for publication and will be formally accepted for publication once it meets all outstanding technical requirements.

Within one week, you’ll receive an e-mail detailing the required amendments. When these have been addressed, you’ll receive a formal acceptance letter and your manuscript will be scheduled for publication.

An invoice for payment will follow shortly after the formal acceptance. To ensure an efficient process, please log into Editorial Manager at http://www.editorialmanager.com/pone/, click the 'Update My Information' link at the top of the page, and double check that your user information is up-to-date. If you have any billing related questions, please contact our Author Billing department directly at authorbilling@plos.org.

If your institution or institutions have a press office, please notify them about your upcoming paper to help maximize its impact. If they’ll be preparing press materials, please inform our press team as soon as possible -- no later than 48 hours after receiving the formal acceptance. Your manuscript will remain under strict press embargo until 2 pm Eastern Time on the date of publication. For more information, please contact onepress@plos.org.

Kind regards,

Dr Thomas Phillips

Staff Editor

PLOS ONE

Journal Requirements:

When submitting your revision, we need you to address these additional requirements.

1. Please ensure that your manuscript meets PLOS ONE's style requirements, including those for file naming. The PLOS ONE style templates can be found at 

https://journals.plos.org/plosone/s/file?id=wjVg/PLOSOne_formatting_sample_main_body.pdf and 

https://journals.plos.org/plosone/s/file?id=ba62/PLOSOne_formatting_sample_title_authors_affiliations.pdf.

2. Please amend your current ethics statement to address the following concerns:

a) Did participants provide their written or verbal informed consent to participate in this study?

b) If consent was verbal, please explain i) why written consent was not obtained, ii) how you documented participant consent, and iii) whether the ethics committees/IRB approved this consent procedure.

3. We note that the grant information you provided in the ‘Funding Information’ and ‘Financial Disclosure’ sections do not match. 

When you resubmit, please ensure that you provide the correct grant numbers for the awards you received for your study in the ‘Funding Information’ section.

Additional Editor Comments:

Thank you for your submission to PLOS ONE. As a Peer-Reviewed Funded Protocol, your submission is eligible for expedited review by in-house editors. Based on our evaluation, we are satisfied that your manuscript reports a protocol for a clinical trial in line with SPIRIT reporting guidelines with a clear statistical analysis plan, which does not deviate from your original trial protocol. Your Study Protocol is therefore considered to be suitable for publication subject to final journal requirements

Reviewers' comments:

# Editor report: Acceptance letter

**DOI:** 10.1371/journal.pone.0261475.r002

**Authors:** Thomas Phillips

10 Dec 2021

PONE-D-21-32625 

Care for capabilities: implementing the capability approach in rehabilitation of patients with neuromuscular diseases. Study protocol of the controlled before-after ReCap-NMD study. 

Dear Dr. Bloemen:

I'm pleased to inform you that your manuscript has been deemed suitable for publication in PLOS ONE. Congratulations! Your manuscript is now with our production department. 

If your institution or institutions have a press office, please let them know about your upcoming paper now to help maximize its impact. If they'll be preparing press materials, please inform our press team within the next 48 hours. Your manuscript will remain under strict press embargo until 2 pm Eastern Time on the date of publication. For more information please contact onepress@plos.org.

If we can help with anything else, please email us at plosone@plos.org. 

Thank you for submitting your work to PLOS ONE and supporting open access. 

Kind regards, 

PLOS ONE Editorial Office Staff

on behalf of

Dr Thomas Phillips 

Staff Editor

PLOS ONE
